# Evolution of synchronous female bilateral breast cancers and response to treatment

**DOI:** 10.1038/s41591-023-02216-8

**Published:** 2023-03-06

**Authors:** Anne-Sophie Hamy, Judith Abécassis, Keltouma Driouch, Lauren Darrigues, Mathias Vandenbogaert, Cecile Laurent, Francois Zaccarini, Benjamin Sadacca, Myriam Delomenie, Enora Laas, Odette Mariani, Thanh Lam, Beatriz Grandal, Marick Laé, Ivan Bieche, Sophie Vacher, Jean-Yves Pierga, Etienne Brain, Celine Vallot, Judicael Hotton, Wilfrid Richer, Dario Rocha, Zakia Tariq, Veronique Becette, Didier Meseure, Laetitia Lesage, Anne Vincent-Salomon, Natalie Filmann, Jenny Furlanetto, Sibylle Loibl, Elise Dumas, Joshua J. Waterfall, Fabien Reyal

**Affiliations:** 1 Department of Medical Oncology, Institut Curie, Université Paris Cité, Paris, France; 2 Residual Tumor & Response to Treatment Laboratory, RT2Lab, Translational Research Department, Paris, INSERM, U932 Immunity and Cancer, Institut Curie, Université de Paris, Paris, France; 3 INRIA, Université Paris-Saclay, CEA, Palaiseau, France; 4 Pharmacogenomics Unit, Department of Genetics, PSL University, Institut Curie, Paris, France; 5 Department of Breast, Gynecological and Reconstructive Surgery, Institut Curie, Université de Paris Cité, Paris, France; 6 Translational Research Department, Institut Curie Research Center, PSL University, Paris, France; 7 INSERM U830, Institut Curie, PSL University, Paris, France; 8 Biological Resource Center, Department of Pathology, Department of Diagnostic and Theranostic Medicine, Institut Curie, PSL University, Paris, France; 9 Department of Gynecology and Obstetrics, Geneva University Hospitals, Geneva, Switzerland; 10 Department of Pathology, Centre Henri Becquerel, INSERM U1245, UNIROUEN, University of Normandie, Rouen, France; 11 INSERM U1016, Faculty of Pharmaceutical and Biological Sciences, Université de Paris Cité, Paris, France; 12 CNRS UMR3244, Institut Curie, PSL University, Paris, France; 13 Department of Surgical Oncology, Institut Godinot, Reims, France; 14 Translational Immunotherapy Team, INSERM U932, Institut Curie, PSL University, Paris, France; 15 Department of Diagnostic and Theranostic Medicine, Institut Curie, University Paris-Sciences et Lettres, Paris, France; 16 German Breast Group, Neu-Isenburg, Germany; 17 Centre for Haematology and Oncology/Bethanien, Frankfurt am Main, Germany

**Keywords:** Breast cancer, Cancer microenvironment, Cancer genomics

## Abstract

Synchronous bilateral breast cancer (sBBC) occurs after both breasts have been affected by the same germline genetics and environmental exposures. Little evidence exists regarding immune infiltration and response to treatment in sBBCs. Here we show that the impact of the subtype of breast cancer on levels of tumor infiltrating lymphocytes (TILs, *n* = 277) and on pathologic complete response (pCR) rates (*n* = 140) differed according to the concordant or discordant subtype of breast cancer of the contralateral tumor: luminal breast tumors with a discordant contralateral tumor had higher TIL levels and higher pCR rates than those with a concordant contralateral tumor. Tumor sequencing revealed that left and right tumors (*n* = 20) were independent regarding somatic mutations, copy number alterations and clonal phylogeny, whereas primary tumor and residual disease were closely related both from the somatic mutation and from the transcriptomic point of view. Our study indicates that tumor-intrinsic characteristics may have a role in the association of tumor immunity and pCR and demonstrates that the characteristics of the contralateral tumor are also associated with immune infiltration and response to treatment.

## Abstract (web summary)

Clinical, genomic and transcriptomic analyses of paired samples of synchronous bilateral female breast cancer identify associations between tumor concordance and immune infiltrates levels and response to neoadjuvant treatment.

## Main

Bilateral breast cancers (BBCs) represent 2–11% of breast cancers^1–3^, and their incidence is increasing owing to advances in breast cancer imaging^4^. This entity includes both synchronous bilateral breast cancers (sBBCs)—that is, occurring synchronously in both breasts—and metachronous bilateral breast cancers (mBBCs)—that is, a tumor occurring in the contralateral breast at a later time period from the primary index cancer. In several studies, sBBCs are associated with poorer survival than unilateral cancer^2,5,6^. Neither synchronous nor metachronous breast cancer is associated with strong genetic determinants, and only 5% of patients with BBC carry *BRCA1* or *BRCA2* mutations^5^.

From the genomic point of view, several studies^7–14^, although with old technologies, investigated clonal relationships among BBCs, with most reaching the conclusion that most, if not all, of BBCs were independent events^7,9,10,13,15–17^. Recently, analyzing a targeted sequencing panel of 254 genes, Begg et al.^18^ investigated the clonality of BBC pairs and found that only one pair of 39 sBBCs was interpreted as clonally related (two shared mutations out of three mutations identified), leaving the question of the independence among sBBCs unresolved.

The immune microenvironment, and especially the role of tumor-infiltrating lymphocytes (TILs), in breast cancer has been studied extensively in the last decade. The drivers of the immunosurveillance of breast cancer derive from both (1) tumor-intrinsic characteristics, such as the subtype of breast cancer, proliferative patterns and tumor mutational burden^19^, and/or (2) extrinsic factors related to the host (for example, sex^20^, age^21^ and body mass index) or the environment (for example, tobacco, alcohol and commensal microbiota). It remains unclear to what extent anti-tumor immunity is driven by the tumor, by the host and/or by the interaction between the host and the tumor.

Neoadjuvant chemotherapy (NAC) is currently administered to patients with locally advanced breast cancers. Molecular subtypes of breast cancers and the density of tumor-infiltrating immune cells are both considered as important predictive and prognostic factors. Many studies have reported associations between high levels of TILs at diagnosis and better response to NAC^22,23^ as well as better prognosis^24^.

sBBCs occur after both breasts have been affected by the same germline genetics, reproductive life factors and environmental exposures for several decades. Two tumors arising concomitantly in a host mimic a model where (1) extrinsic factors are almost fully shared by the same host; (2) intrinsic factors are specific to the tumor of each side; and (3) the immune tumoral microenvironment resulting from the interaction between the same host and two different tumors can be compared.

In the current study, we identified a rare resource of 20 tumors deriving from six patients with sBBCs treated by NAC with left and right pre-NAC and post-NAC with frozen material available. We performed whole-exome sequencing (WES), copy number alterations (CNAs) and RNA sequencing (RNA-seq) to comprehensively analyze somatic alterations, the immune microenvironment and the tumor evolution under treatment.

## Results

### Patient and tumor characteristics

**Extended Data Fig. 1:**
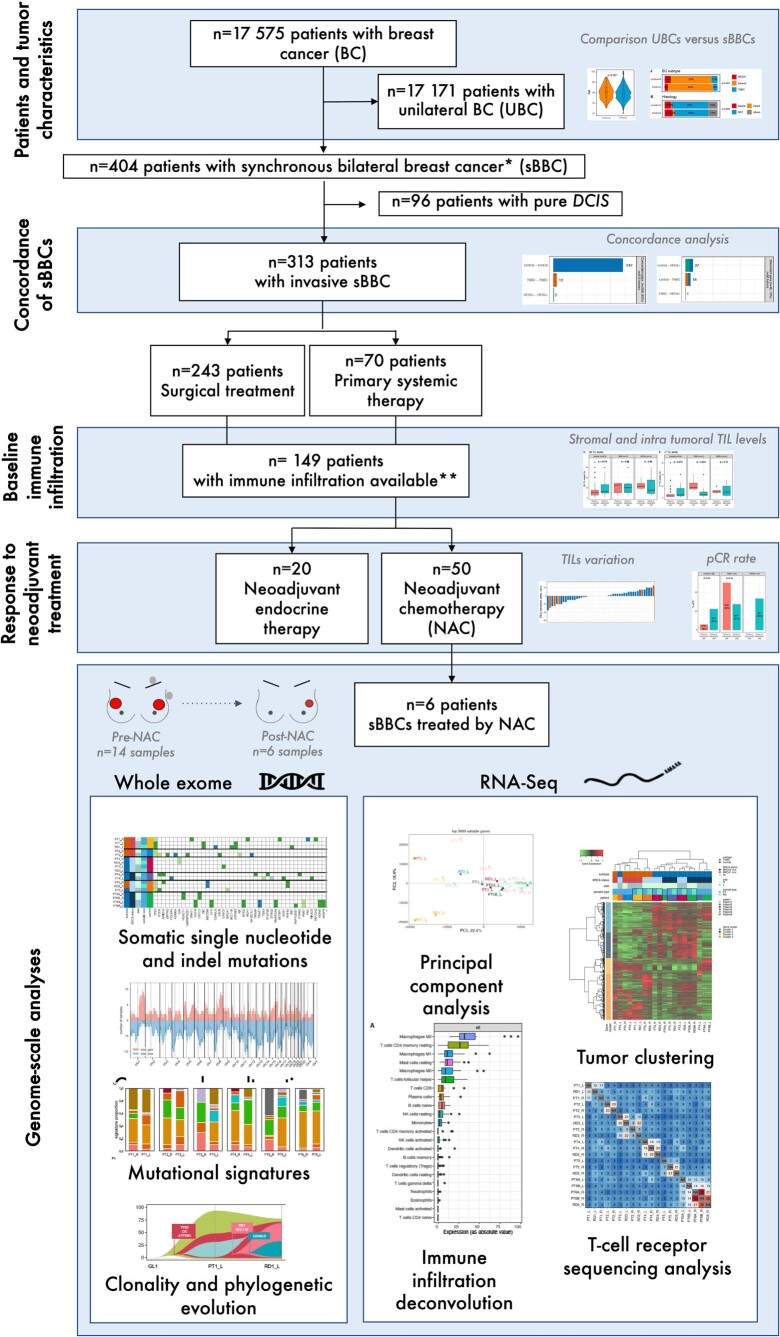
Flow chart of the study cohort. Abbreviations: DCIS: Ductal carcinoma in situ; NAT: neoadjuvant treatment; NAC: neoadjuvant chemotherapy; pCR: pathological complete response; RNAseq, RNA sequencing; sBBC, synchronous bilateral breast cancers; TIL, tumor infiltrating lymphocytes; UBC: unilateral breast cancer; WES, whole exome sequencing.

Out of 17,575 patients with breast cancer in our institutional clinical database, 404 patients had sBBCs (2.3%) (Extended Data Fig. 1). Slight differences existed in patient and tumor characteristics between patients with unilateral breast cancers and patients with sBBCs (Supplementary Table 1 and Supplementary Fig. 1). Out of 313 patients with invasive sBBCs, most of the tumors were luminal (*n* = 538, 87.6%), whereas triple-negative breast cancer (TNBC) (*n* = 44, 7.2%) and *HER2*^+^ breast cancers (*n* = 32, 5.2%) were rare (Supplementary Table 2). Only 13 patients were carriers of a genetic germline *BRCA1* or *BRCA2* predisposition. They were significantly younger and more likely to be diagnosed with large, palpable and high-grade tumors, more frequently of TNBC subtype (Supplementary Table 3).

### Concordance of sBBCs

**Fig. 1:**
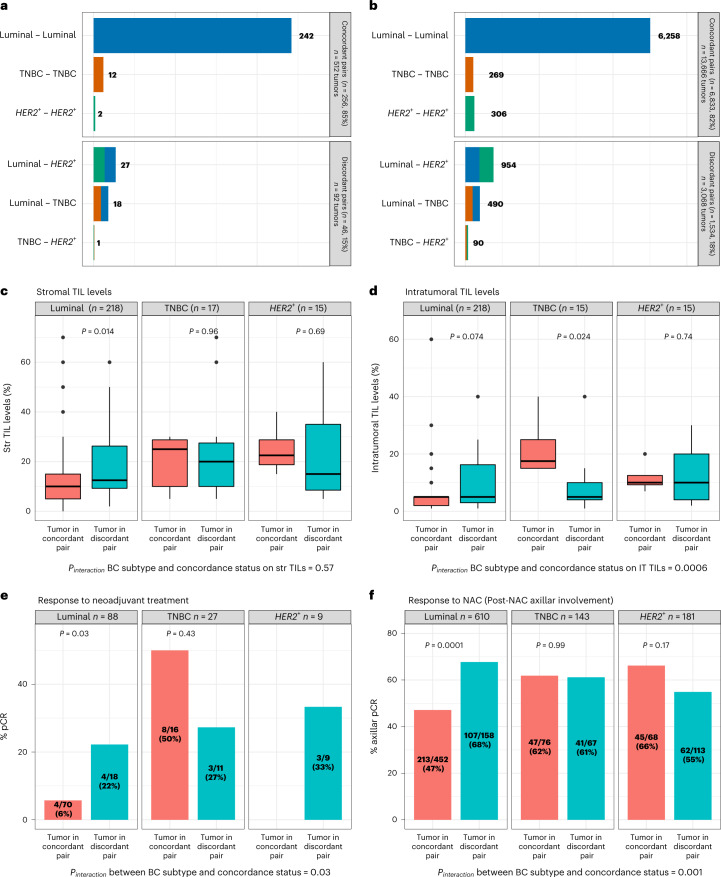
Tumor characteristics of the pairs of sBBCs included in the cohort. **a**, Repartition of the association of the subtypes of breast cancers within a pair of sBBCs according to the concordance or the discordance status of the pair in the Curie cohort; tumor characteristics are based on the 302 pairs with concordance subtype available out of 313 pairs. The concordance refers to the status of both tumors within a pair of sBBCs regarding the subtype of breast cancer, either of the same subtype of breast cancer (tumor in concordant pair) or of different subtypes of breast cancers (tumor in discordant pair). **b**, Repartition of the association of the subtypes of breast cancers within a pair of sBBCs according to the concordance or the discordance status of the pair in the SEER cohort (8,367 patients with sBBCs, *n* = 16,734 tumors). **c**, Stromal TIL levels of the index tumor by subtype of breast cancer and by the concordance status of the pair it belongs to. Lower and upper bars represent the first and third quartiles, respectively; the medium bar is the median; and whiskers extend to 1.5 times the IQR. **d**, Intratumoral TIL levels of the index tumor by subtype of breast cancer and by the concordance status of the pair it belongs to. Lower and upper bars represent the first and third quartiles, respectively; the medium bar is the median; and whiskers extend to 1.5 times the IQR. **e**, pCR rates of the index tumor by subtype of breast cancer and by the concordance status of the pair it belongs to. **f**, Axillar pCR rates (proportion of patients with a post-NAC number of positive nodes >1 divided by the total number of patients) of the index tumor by subtype of breast cancer and by the concordance status of the pair it belongs to (*n* = 467 patients treated with NAC, representing 934 tumors). Statistical tests were Wilcoxon tests (**c**,**d**), Fisher tests (**e**) and chi-square tests (**f**). IT, intratumoral; Str, stromal.

Overall, the 313 paired invasive sBBC tumors shared more common characteristics than expected by chance (Supplementary Table 4): most (84.7%) of the tumor pairs were concordant regarding clinical and pathological patterns, notably regarding the subtype of breast cancer (Fig. 1a). A minority of pairs of tumors belonged to different breast cancer subtypes (discordant pairs: 15.3%), and both the proportion of pairs (18%) and their relative repartition were similar in the validation cohort of 8,367 patients with sBBCs from the Surveillance, Epidemiology, and End Results (SEER) database (Fig. 1b).

### Baseline immune infiltration and variation after neoadjuvant treatment

**Extended Data Fig. 2:**
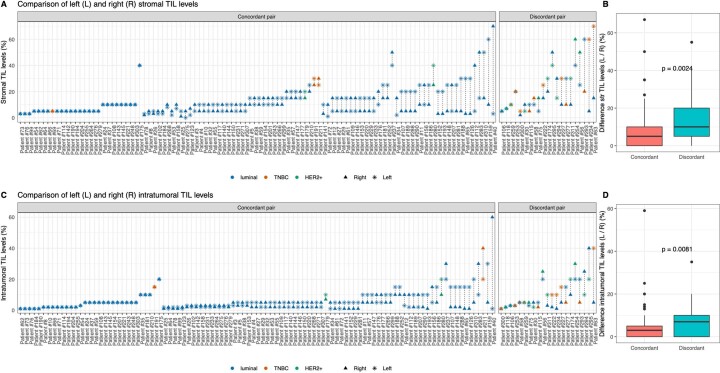
Comparison of the left and the right immune infiltration of pairs of sBBCs according to the BC subtype and the concordance status of the tumor pair. The cohort comprises 125 patients (n = 250 tumors) sBBCs and both immune infiltration and concordance status of the pair available (n = 125 and 248 respectively for intratumoral TILs). A. stromal TIL levels; B. Comparison of the absolute difference of between the stromal TIL levels of the left and the right side; Lower and upper bars represent the first and third quartile, respectively, the medium bar is the median, and whiskers extend to 1.5 times the inter-quartile range. C. Intratumoral TIL levels; D. Comparison of the absolute difference of between the intratumoral TIL levels of the left and the right side. Lower and upper bars represent the first and third quartile, respectively, the medium bar is the median, and whiskers extend to 1.5 times the inter-quartile range. Statistical tests were Wilcoxon tests (Extended Data Fig. 2B, D) and are two-sided. Abbreviations: str TIL, stromal tumor infiltrating lymphocytes; TNBC: triple negative breast cancer.

Immune infiltration levels before treatment were assessed by the presence of a mononuclear cell infiltrate, following the recommendations of the international TILs Working Group^25,26^, on hematoxylin-and-eosin (H&E)-stained sections in 149 patients (277 tumors). The difference between the TIL levels from the left and the right tumor was higher in pairs of discordant subtypes of breast cancers than in pairs of concordant subtypes (Extended Data Fig. 2). At the tumor level, TIL levels were independently associated with higher-grade tumors (Supplementary Tables 5 and 6), and, interestingly, the relationship between TIL levels and the subtype of breast cancer showed a systemic effect—that is, it was affected by the subtype of the contralateral tumor. In luminal breast cancers, stromal TIL levels were lower when the subtype of the contralateral tumor was concordant than when it was discordant, and the same trend was observed for intratumoral TILs (Fig. 1c,d). Conversely, in TNBCs, the intratumoral TIL levels were lower when the subtype of the contralateral tumor was concordant than when it was discordant. The interaction test was highly significant (*P*_*interaction*_ = 0.0006), indicating that the impact of tumor subtype on intratumoral immune infiltration was significantly modified by the concordance of the subtype of breast cancer of the tumor pair it belonged to. This result was also validated in a third independent cohort from the German Breast Group (GBG), where the interaction tests were highly significant both for stromal and intratumoral TILs (*P*_*interaction*_ = 0.007 and *P*_*interaction*_ = 0.006 respectively) (Supplementary Fig. 2). This suggests that TIL levels are not determined purely by local tumor microenvironment properties.

**Extended Data Fig. 3:**
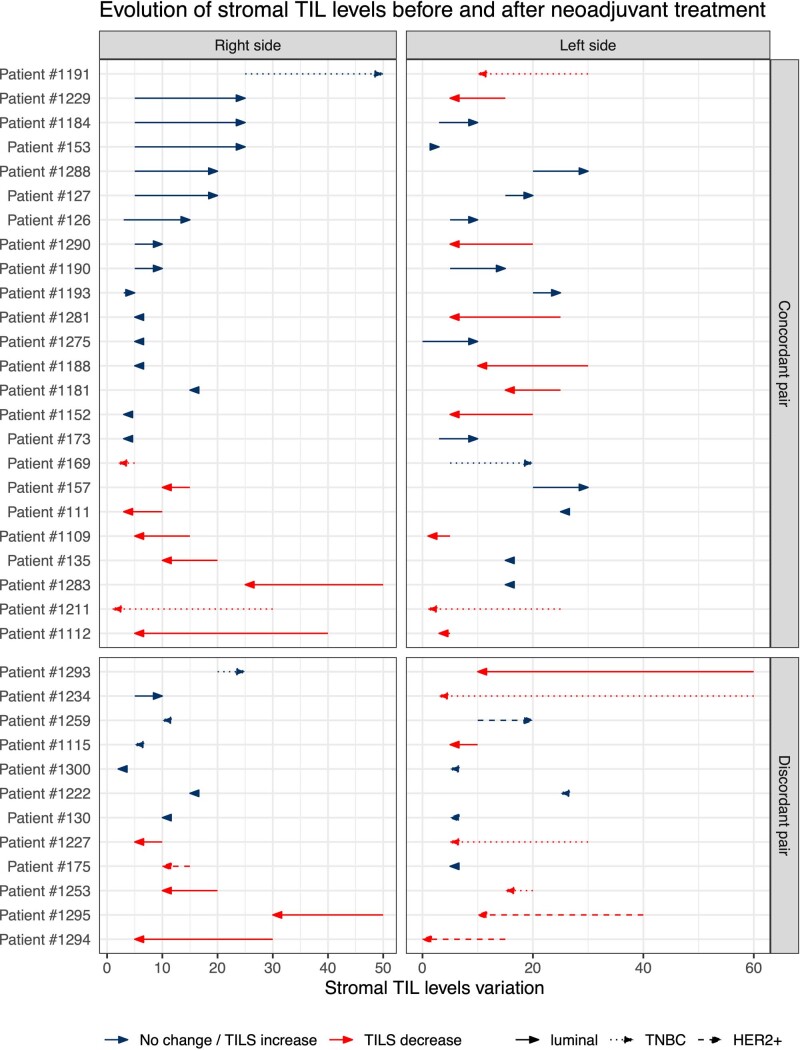
Comparison of the pre and post-treatment stromal TIL levels of pairs of sBBCs according to the BC subtype and the concordance status of the tumor pair. The cohort comprises 37 patients (n = 74 tumors) with sBBCs treated with neoadjuvant treatment with both immune infiltration and concordance status of the pair available. Abbreviations: NAT: neoadjuvant treatment; str TIL, stromal tumor infiltrating lymphocytes; TNBC: triple negative breast cancer.

**Extended Data Fig. 4:**
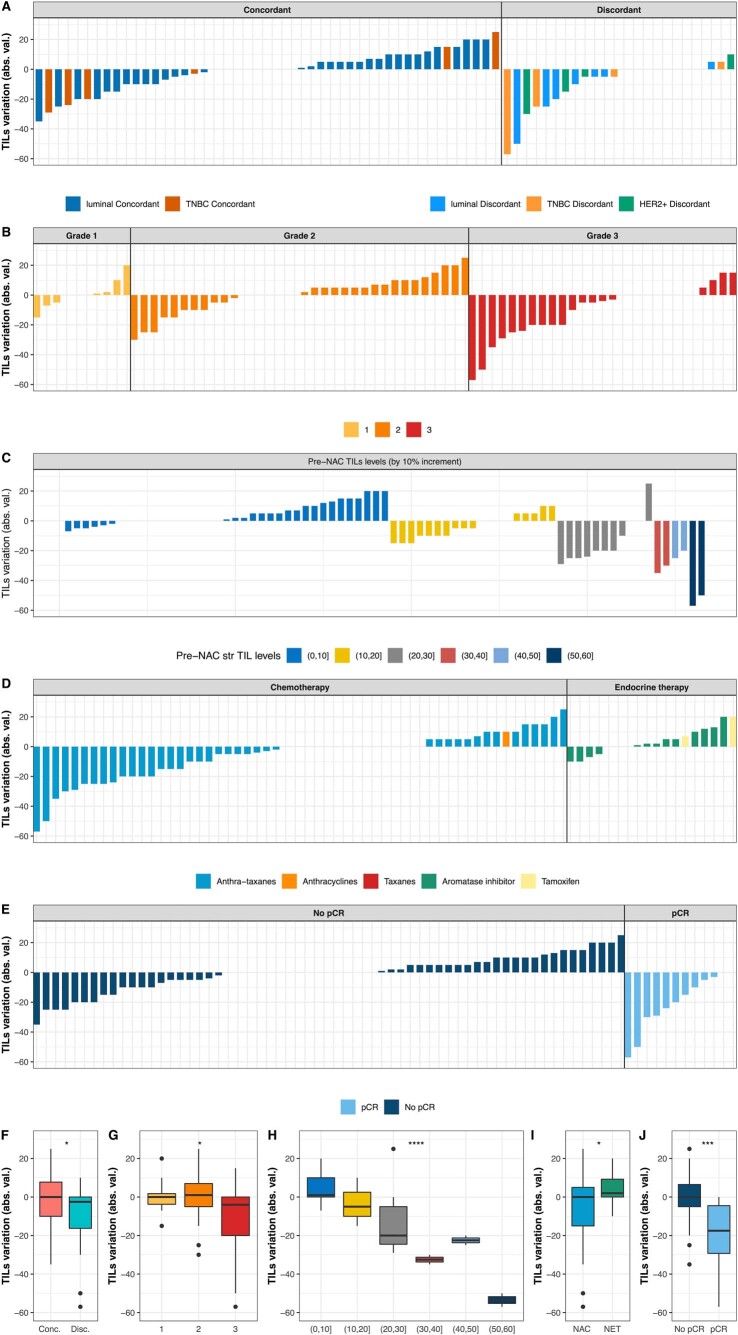
Stromal TIL levels’ variation before and after neoadjuvant treatment (NAT). The cohort comprises 37 patients (n = 74 tumors) with sBBCs treated with neoadjuvant treatment with both immune infiltration and concordance status of the pair available. A. Waterfall plot representing the variation of stromal TIL levels according to BC subtype and to the discordant or concordant status of the pair regarding tumor type; Each bar represents the absolute TIL variation, that is, the difference between stromal TIL levels after and before NAT; each bar represents one sample, and samples are ranked by increasing order of TIL level change. Paired samples for which no change was observed have been left on the graph; B. according to the tumor grade; C according to the pre-NAT TILs levels, binned by increments of 10%. Within each pre-NAC TIL levels category, the change in TIL levels is ranked by increasing TIL level variation; D. According to the type of neoadjuvant treatment; E. According to the pCR status; Box plots of the association between TILs variation (as absolute value) and: F. concordant or discordant status of the pair; G. tumor grade; H pre-NAT TIL levels by 10% increment I: type of treatment; J pCR status. In boxplots, lower and upper bars represent the first and third quartile, respectively, the medium bar is the median, and whiskers extend to 1.5 times the inter-quartile range. Statistical tests were Wilcoxon tests and are two-sided. Stars represent *p*-values for t-test: *: 0.01 ≤ p ≤ 0.05; **: 0.001 ≤ p < 0.01; ***: p < 0. 001.Exact *p*-values for concordance regarding BC subtype, tumor grade, pre-NAT TILs, type of neoadjuvant treatment, pCR status are p = 0.027, p = 0.024, p = 6.1 e10-6, p = 0.017, p = 0.0002, Abbreviations: Conc.: tumor belonging to a concordant tumor pair; Disc.: tumor belonging to a discordant tumor pair; NAT: neoadjuvant treatment; NAC: neoadjuvant chemotherapy; NET: neoadjuvant endocrine therapy; str TIL, stromal tumor infiltrating lymphocytes; TNBC: triple negative breast cancer.

On paired pre-neoadjuvant treatment (NAT) and post-NAT data on immune infiltration available for 74 tumors (37 patients) (Extended Data Fig. 3), stromal TIL levels decreased in 30 tumors (40.5%), remained stable in 18 tumors (24.3%) and increased in 26 tumors (35.1%). The decrease of TIL levels was of larger magnitude in tumors belonging to discordant pairs, to higher tumor grade and with high pre-NAC stromal TIL levels, and, in case of treatment with NAC rather than NET, the TIL decrease was very strongly associated with the occurrence of a pathologic complete response (pCR) (Extended Data Fig. 4). As a whole, stromal TIL levels were not significantly different before and after NAT, but pre-NAT and post-NAT stromal TIL levels were significantly different according to the type of NAT in discordant, grade 3 tumors and in tumors that reached pCR (Supplementary Fig. 3). These findings suggest that NAT reshapes the immune contexture of sBBCs.

### Response to NAT

Twenty-two tumors out of 140 tumors reached pCR. Pre-NAT stromal TIL levels and the subtype of breast cancer were independently associated with the occurrence of a pCR (Supplementary Table 7). As was seen for TIL levels, the pCR rates showed a systemic effect when the contralateral tumor subtype was discordant. In luminal breast cancers, the pCR rate was significantly higher when the contralateral pair was of discordant subtype (22% versus 6%), whereas no such pattern was found in the other subtypes (*P*_*interaction*_ = 0.03) (Fig. 1e). Similar results were found in two independent validation cohorts. In the SEER validation cohort, the difference in the rate of axillar pCR in tumors belonging to discordant pairs versus in tumors belonging to concordant pairs was highly significant (68% versus 47%, *P* = 0.00001, respectively) (Fig. 1f). In the GBG cohort, the pCR rate in luminal breast cancers was significantly higher in tumors belonging to discordant pairs versus in tumors belonging to concordant pairs (30% versus 6%, *P* = 0.0002, respectively) (Supplementary Fig. 4). Survival analyses showed that clinical T stage, breast cancer subtype and tumor grade were significantly associated with relapse-free survival (RFS) (Supplementary Table 8).

### Genome-scale analyses on pre-NAC and post-NAC samples from six patients

**Extended Data Fig. 5:**
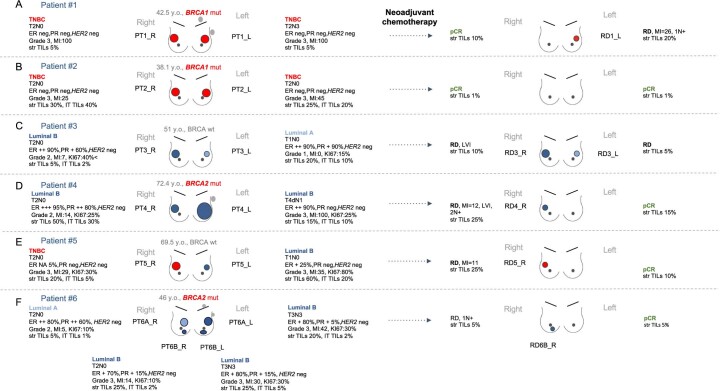
Patient’s and tumor’s characteristics and response to neoadjuvant chemotherapy in the 6 patients (20 samples) with NGS data. Primary tumors are labelled PT, specimens with residual disease are labelled RD. Abbreviations: ER, estrogen receptor; intratumoral TIL, intratumoral tumor infiltrating lymphocytes; L, left; neg, negative; pCR, pathologic complete response; PR, progesterone receptor; PT, patient; R, right, RD, residual disease; str TIL, stromal tumor infiltrating lymphocytes; TNBC: triple negative breast cancer.

Of 50 patients with sBBCs treated with NAC, frozen material of sufficient quality was available in six patients to perform tumor/normal WES and tumor RNA-seq in both left and right pre-NAC and post-NAC samples (including one patient with a multicentric bilateral breast cancer) (Extended Data Fig. 5 and Supplementary Table 9). The total number of samples was 20 (pre-NAC: *n* = 14; post-NAC: *n* = 6), and this cohort was further used for all the genome-scale analyses, both at the DNA level and the RNA level. Germline pathogenic mutations in breast cancer predisposition genes were identified in four patients (*BRCA1*, *n* = 2; *BRCA2*, *n* = 2). Among the 14 primary tumors (PTs), nine were of luminal subtype, and five were TNBCs. All patients received standard sequential anthracyclines/cyclophosphamide followed by taxanes. After NAC completion, six out of 14 tumors had residual disease (RD), whereas eight tumors reached pCR.

#### Somatic single-nucleotide indel mutations

**Fig. 2:**
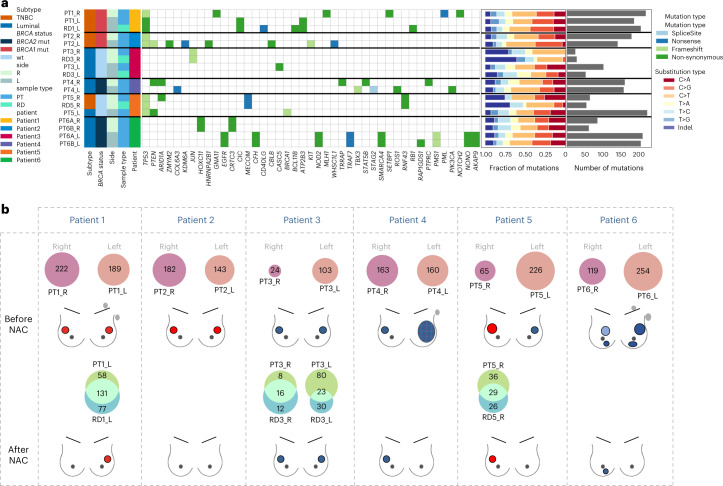
Tumor mutation profiles. The analyses were performed in the left, right, pre-NAC (PT) and post-NAC (RD) samples of a cohort of six patients (20 samples) with next-generation sequencing data available. **a**, Heat map of somatic driver mutations (including missense, nonsense and splicing) detected in six study patients and 20 samples. **b**, Venn diagrams showing the number of mutations shared between the left (pink) and the right (purple) primary tumors of a same patient and shared (intersect turquoise) between the PT (light green) and the corresponding specimen after NAC (RD, blue). The post-NAC samples RD4_R and RD6B_R were discarded owing to poor sample purity. Mutations from the two multicentric tumors of each side of patient 6 were merged. L, left; R, right; wt, wild-type.

Twenty tumor samples were profiled by WES and RNA-seq, and distant juxta-tumor samples were used for germline WES sequencing in each patient. A median of 151.5 somatic mutations were detected per tumor, and a median of three mutations were annotated as potential drivers in each sample (Supplementary Table 10). No mutation was shared between the left and right side of the PTs from any patient, consistent with the contralateral tumors developing from independent clones (Fig. 2). Most of the mutations were shared between a PT sample and the matched RD.

#### Neoantigens

We predicted potential neoantigens from somatic mutations using netMHCpan^27^ after determining human leukocyte antigen (HLA) haplotypes with Seq2HLA^28^. Most of the antigens were predicted from HLA-C, and the repartition of predicted neoantigens was evenly distributed across patients (Supplementary Fig. 5a). The number of neoantigens was positively correlated with the levels of stromal TILs (Supplementary Fig. 5b), was not associated with the breast cancer subtype (Supplementary Fig. 5c) and was higher in PT samples than in RD samples (Supplementary Fig. 5d). No neoantigen was shared across patients, and no neoantigen was shared between the left and the right tumors. Conversely, the RD shared most of the mutations with the corresponding PT (Supplementary Fig. 5e).

#### CNAs

**Fig. 3:**
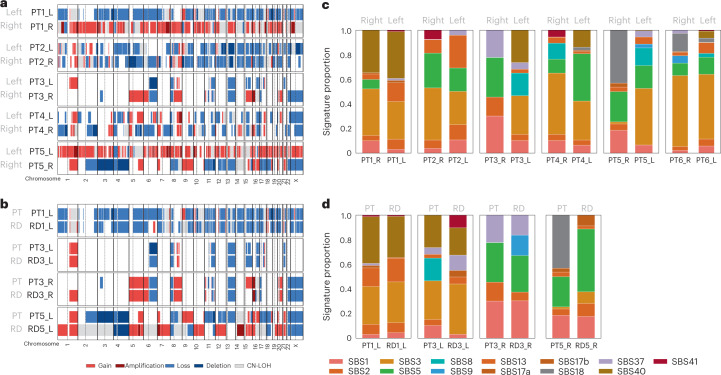
CNAs and mutational signatures profiles. The analyses are performed in the left, right, pre-NAC and post-NAC samples of a cohort of six patients (20 samples) with next-generation sequencing data available. Mutational signatures published by Alexandrov^29^ were calculated using the deconstructSigs package. **a**, CNAs are compared among the two sides of the primary tumor of the same patient. **b**, Among the primary tumor and its corresponding sample with RD after NAC. **c**, Mutational signatures are compared among the two sides of the primary tumor of the same patient (mutations from the two multicentric tumors of each side of patient 6 have been merged). **d**, Among the primary tumor and its corresponding sample with RD after NAC. CN-LOH, copy-neutral loss of heterozygosity.

Copy number analysis of the WES profiles identified recurrent gains (eight out of 12 samples) at 1q, 8q and 17q and recurrent losses (over 50% of the samples) at 4p, 8p, 6q, 13q, 16q and, to a lesser extent, 1p (Supplementary fig. 6). Most of the alterations were not shared between the left and the right side (Fig. 3a), whereas most of the CNAs were consistent between PT and RD (Fig. 3B) (mean cosine distance 0.25 versus 0.75, *P* = 0.03; Supplementary Fig. 7).

#### Mutational signatures

We analyzed mutational signatures by deconvoluting the frequency of the 96 different possible trinucleotide substitutions against known signatures of mutation patterns^29^ (Fig. 3c). Similarities regarding mutational processes were lower within the left and right side of the PTs than within pairs of PT–RDs (Fig. 3d).

#### Clonality and phylogenetic evolution

**Fig. 4:**
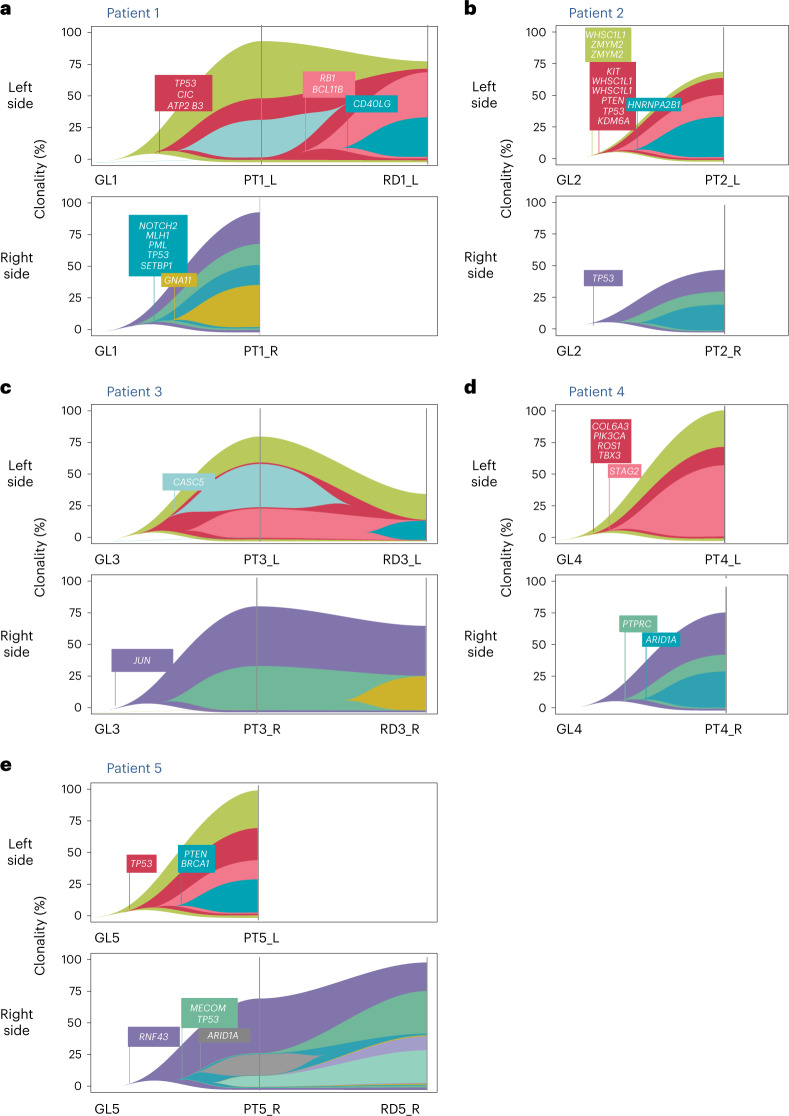
Fish plot retracing phylogeny between left and right PTs and corresponding RD. Each subfigure represents the evolution of the tumors of a given patient under NAC. The upper fish plot represents the evolution of the left tumor(s); the lower fish plot represents the evolution of the right tumor(s). Each fish plot displays the prevalence of subclones throughout treatment. Subclonal architecture was reconstructed with SuperFreq. Subclonal profiles show annotated common driver cancer genes. **a**, Patient 1: Both PTs were mutated for P53, but the genomic alteration was different on the left and the right side (right side, indel frameshift deletion position 7578213; left side, substitution C>T p.R175C missense substitution identified as pathogenic in ClinVar and present in the RD). **b**, Patient 2: Both tumors were mutated for TP53 with two different mutations (right side, frameshift loss of a nucleotide position 7577558). **c**, Patient 3. **d**, Patient 4: The right sample with RD (RD4_R) was discarded from analysis owing to low purity. **e**, Patient 5: TP53 was mutated on both sides (left side, frameshift deletion position 7578213; right side, frameshift deletion position 7579522).

We determined the phylogenetic evolution between the germline profile to the left and right primary tumors and ultimately to the RD if present (Fig. 4a–e). Genomic profiling found no common clones between bilateral PTs of the same patient, showing that these tumors arose through unrelated tumor evolution processes.

Altogether, these results suggest that left and right PTs are not clonally related and that their evolution under NAC does not converge to a common profile. Hence, RD is closer to its associated PT than to its concomitant contralateral tumor.

#### Independent validation cohort of sBBCs for WES analyses

**Extended Data Fig. 6:**
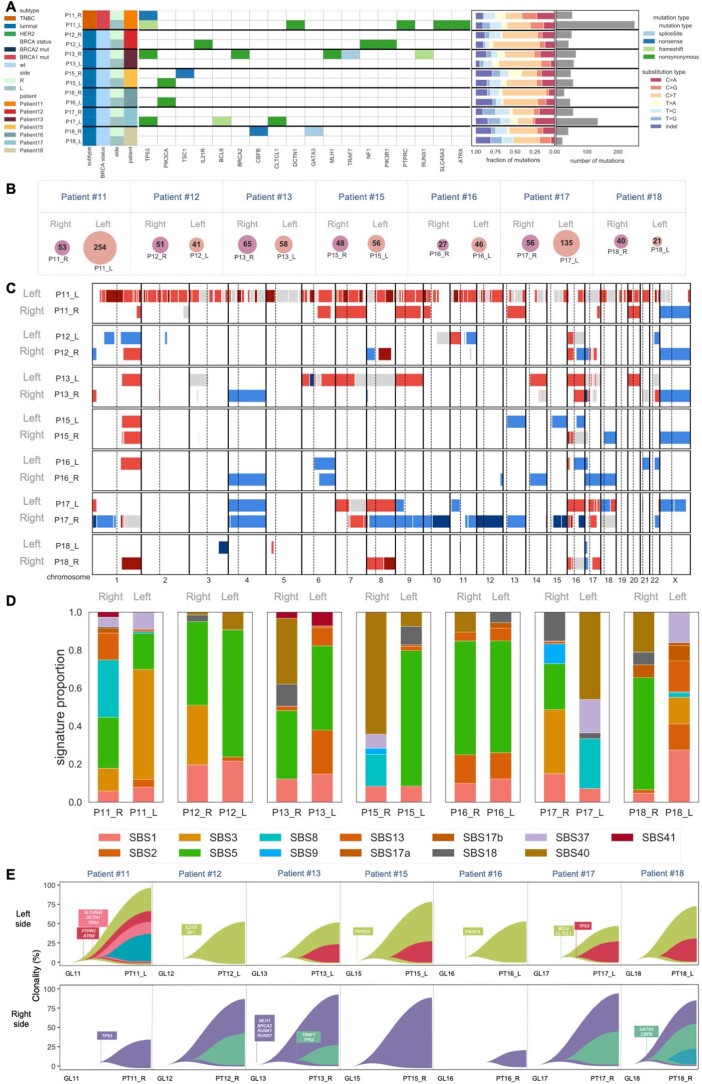
Genomic profiles of 14 tumors from 7 pairs of sBBCs in an in-house independent validation cohort of patients treated by surgery as first treatment. A. Tumor mutation profiles: Heatmap of somatic driver mutations (including missense, nonsense, and splicing); B. Venn diagrams showing the number of mutations shared between the left (pink) and the right (purple) primary tumors of a same patient; C: Copy number alterations (CNA); D: Mutational signatures profiles as published by Alexandrov^25^ are calculated using the deconstructSigs package.; E: Fishplots retracing phylogeny between left, right primary tumors. Abbreviations: L, left; R, right; RD, TNBC, triple negative breast cancer.

We performed WES analyses from an independent validation cohort of 14 sBBC samples treated by surgery as first treatment. Similar results were found regarding the genomic profiles of the left and right tumors: left and right tumors were found to be genomically independent in terms of mutations (Extended Data Fig. 6a,b), CNAs (Extended Data Fig. 6c), mutational signatures (Extended Data Fig. 6d) and phylogenetic evolution (Extended Data Fig. 6e).

#### Particular case of multicentric tumors

**Fig. 5:**
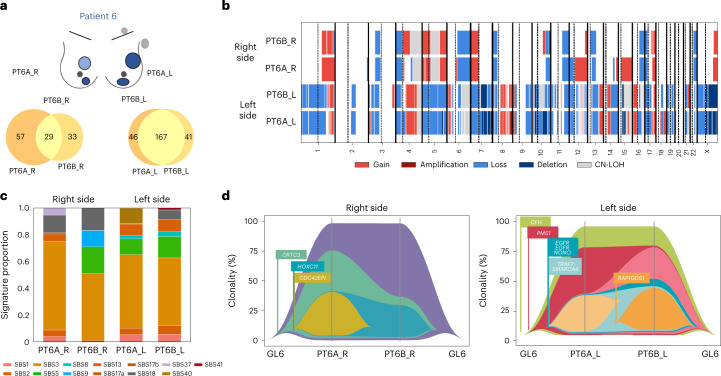
Genomic alterations in the patient with multicentric sBBCs. The analyses were performed in the two left tumors (PT6A_L and PT6B_L) and in the two right tumors (PT6A_R and PT6B_R) of the same patient (patient 6). The sample with RD (RD6B_R) was discarded owing to poor sample purity. **a**, Tumor mutation profiles: Venn diagrams showing the number of mutations shared between the two primary tumors of each side (yellow intersect). **b**, CNA profiles, compared between the two multicentric tumors of each side. **c**, Mutational signatures as from Alexandrov^29^ compared between the two multicentric tumors of each side. **d**, Fish plot retracing the phylogeny between the two multicentric tumors of each side. Each fish plot can be interpreted from the left to the right or from the right to the left, corresponding to the emergence or the extinction of a clone, respectively. CN-LOH, copy-neutral loss of heterozygosity.

One patient had a bilateral multicentric tumor (patient 6) in the context of a *BRCA2* pathogenic germline mutation. Although the left and the right tumors shared no common mutations, the two tumors from each side shared most genetic alterations at both the substitution (Fig. 5a) and copy number (Fig. 5b) levels as for mutation signature analyses (Fig. 5c). On each side, phylogenetic reconstruction clearly indicated that multicentric tumors were clonally related, with one tumor evoluting to a neighboor tumor through the extinction/emergence of particular subclones.

### Transcriptomic alterations

#### Tumor clustering and principal component analysis

**Fig. 6:**
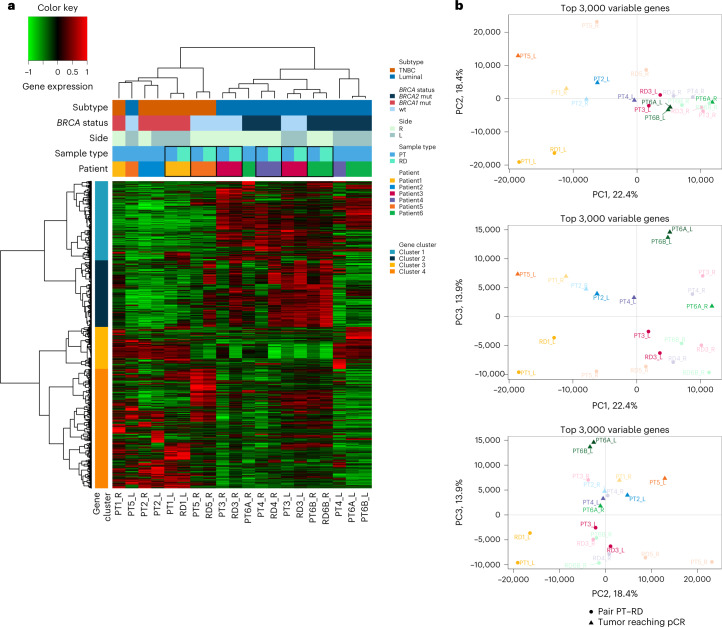
Transcriptomic analyses of the sBBC cohort. **a**, Gene expression clustering with RNA-seq data based on the 2,846 most variant genes on the left, right, pre-NAC and post-NAC samples of a cohort of six patients (20 samples) with RNA-seq data available. Cluster 1 is enriched in genes coding for early and late response to estrogens; cluster 2 is enriched in genes coding for TNF signaling, myogenesis and epithelial–mesenchymal transition; cluster 3 is enriched in genes coding for G2M checkpoints, E2F targets and cellular cycle; and cluster 4 shows no clear enrichment in specific pathways. **b**, PCA using the 3,000 most variable genes. L, left; PC, principal component; R, right; wt, wild-type.

We performed unsupervised hierarchical clustering based on transcriptomic profile of the most variable genes, and gene clustering split the 2,846 genes into four main clusters (Fig. 6a). The PT samples consistently clustered with their related RD rather than the tumor from the contralateral side. Similar results were seen after principal component analysis (PCA) using the 3,000 most variable genes (Fig. 6b). This suggests that PT and RD are closer from a transcriptomic point of view than are left and right tumors from the same patient.

#### Qualitative immune infiltration analysis with deconvolution and T cell receptor sequencing analysis

**Extended Data Fig. 7:**
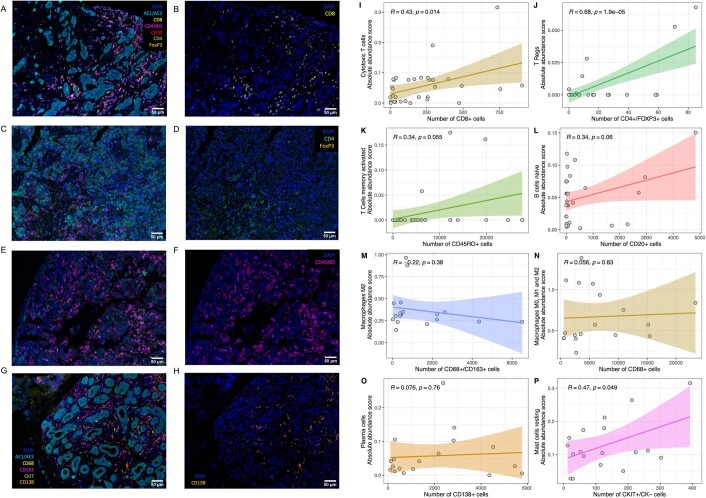
Multiplex immunofluorescence (IF) images and their correlation with abundance of the immune subset inferred by CIBERSORT. The stainings were performed in the in left, right, pre and post-NAC samples of a cohort of 6 patients (20 samples). The images A to H show the same regions of tumors stained with the whole panel (left facet) and focusing on an antibody (right facet). Markers of the panel #1 (Figures A to F) include - CD4 – CD8 – CD45RO – FOXP3 – CD20 – AE1/AE3, and markers of the panel #2 (Figures G-H) include CD68 – CD 163 – CD138 – CKIT – AE1/AE3. We used the total number of stained cells for an antibody to perform the correlation with immune subsets inferred by the deconvolution, with the following correspondence: CD8 + cells: Cytotoxic T cells; CD4 + /FOXP3 + cells: T regs; CD45RO + cells: T cells memory activated; CD20 + cells: B cells naïve; CD68 + /CD163 + cells: Macrophages M2; CD68 + cells: Macrophages M0,M1, and M2; CD138 + cells: Plasma cells. Image size is 0.67 mm×0.5 mm, and resolution is 0.4999 μm /pixel (x20). The two RD samples could not be assessed in patient #3 due to material exhaustion. Sample of primary tumor in patient #6 right tumor (tumor A) stained with the whole panel #1; B. with a highlight on CD8 + cells; C. Sample of primary tumor in patient #5 left. tumor stained with the whole panel #1; D. with a highlight on CD4 + /FOXP3 + cells; E. Sample of primary tumor in patient #2 right tumor stained with the whole panel #1; F. highlight on CD45RO + cells; G. Sample of primary tumor in patient #4 right tumor stained with the whole panel #2; H. with a highlight on CD138 + cells. Spearman correlation coefficient between the number of stained cells with one antibody, and the proportion of the corresponding immune subtype as inferred by deconvolution applied to the gene expression data; I:Absolute abundance score of cytotoxic T cells by number of CD8 + cells; J: Absolute abundance score of T Regs cells by number of CD4 + /FOXP3 + cells; K: Absolute abundance score of Memory T cells by number of CD45RO + cells; L: Absolute abundance score of B cells naïve by number of CD20 + cells; M: Absolute abundance score of Macrophages M2 by number of CD68 + /CD163 + cells; N: Absolute abundance score of Macrophages M0,M1, and M2 by number of CD68 + cells; O: Absolute abundance score of plasma cells by number of CD138 + cells; P: Absolute abundance score of Mast cells resting by number of cKIT + /CK- cells. Statistical tests were Pearson correlation coefficients (Extended Data Fig. 7I-P). The lines represent the fitted linear regression line and the colored bands represent the 95% confidence interval bands. The staining was performed once for every case. White scale (bottom right) represents 50 µm.

**Extended Data Fig. 8:**
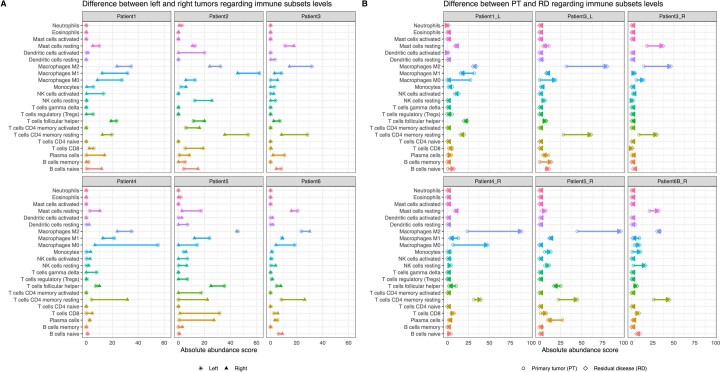
Comparison of the repartition of the subsets of immune subpopulation in the left and the right tumors of pairs of sBBCs and in the pre and post NAC pairs of sBBCs. The analyses are performed in the in left, right, pre and post-NAC samples of the cohort of 6 patients (20 samples). Each subfigure represents the amount of immune subpopulation evaluated with the CIBERSORT algorithm; A. in the right tumor (triangle) and the paired left tumor (star). Both tumors of each side of patient 6 are averaged, as they show very minimal differences in the composition of the immune microenvironment; B in the primary tumor (PT) (circle) and the paired residual disease (RD) (diamond). Abbreviations: PT, primary tumor; RD, residual disease.

**Extended Data Fig. 9:**
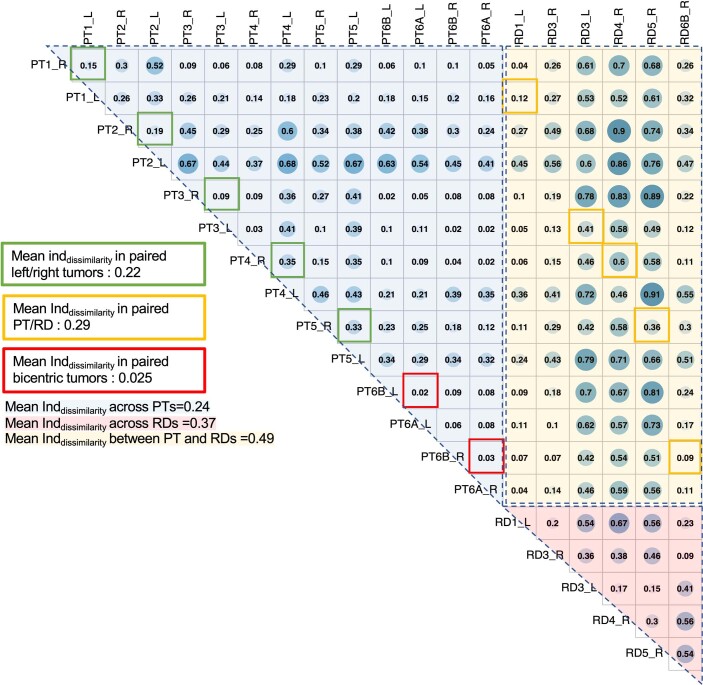
Comparison of the similarity of the immune microenvironnement across samples. The analyses are performed in the in left, right, pre and post-NAC samples of the cohort of 6 patients (20 samples). The composition of the immune microenvironment (22 immune subpopulations) was deconvoluted by the CIBERSORT algorithm (absolute mode) as described by Newman^26^. A dissimilarity index of the immune microenvironnement was displayed sample *versus* sample. The colored area represents the samples compared at the cohort levels (dissimilarity among the PT samples (blue area), among the RD samples (orange area), and between PTs samples compared with RDs samples (yellow area). The colored bordered squares represent the comparison of paired data (samples of the same side of a patient (red bordered squares), the left and the right sides (green bordered squares), and between PT and related RDs (yellow bordered squared). L, left; PT, primary tumor; R, right; RD, residual disease.

We applied the CIBERSORT algorithm^30^ using the ‘absolute’ mode to deconvolute RNA-seq expression profiles into 22 subsets of immune subpopulations on the 20 samples of the cohort. The top three most abundant immune subpopulations were M2 macrophages, CD4 memory resting T cells and M1 macrophages (Supplementary Fig. 8a). CD4 memory T cells and M2 macrophages were increased in RD compared to PT (Supplementary Fig. 8b). Immunofluoresence was performed to evaluate the concordance between the immune subsets assessed by deconvolution and orthogonal techniques. The correlation coefficient between both metrics was statistically significant regarding cytotoxic T cells (CD8^+^ cells), Tregs (CD4^+^/FOXP3^+^ cells) and Mast cells resting (CKIT^+^/CK^−^ cells) (Extended Data Fig. 7i,j,q); was nearly significant for T cells memory activated (CD45RO^+^ cells) and B cells naive (CD20^+^) (Extended Data Fig. 7k,l); and was not significant in macrophages M2 (CD68^+^/CD163^+^ cells) or macrophages of any type (CD68^+^ cells) and plasma cells (CD138^+^ cells) (Extended Data Fig. 7m–o). At the patient level, the immune composition of the paired contralateral tumors was different regarding several immune subsets (macrophages M0, M1 and M2 and T cells CD4 memory activated and resting) (Extended Data Fig. 8a), whereas the variation of the immune composition between PT and RD mostly concerned increasing levels of macrophages M2 and M0 and T cells CD4 memory resting (Extended Data Fig. 8b). We compared the predicted immune contexture patterns among samples of the cohort using a dissimilarity index (I_dissimilarity_). The higher the dissimilarity index, the more the composition of the immune infiltration differs. Neither the mean dissimilarity indices (Extended Data Fig. 9) of paired left and right tumor (green-bordered squares, mean I_dissimilarity_ = 0.22) nor paired PT and RD (yellow-bordered squares, mean I_dissimilarity_ = 0.29) were statistically different from the rest of the samples. At the cohort level, the dissimilarity was lower among the PT samples (blue area) than among the RD samples (orange area) (mean I_dissimilarity_: 0.24 versus 0.37, *P* = 0.009), whereas the greatest difference was seen between PT samples compared to RD samples (yellow area, mean I_dissimilarity_ = 0.49). These results suggest that the composition of the immune microenvironment is strongly associated with the pretreated or non-pretreated character of the sample (PT or RD), in line with the changes in the immune contexture induced by NAC treatment.

### T cell receptor sequencing analysis

**Extended Data Fig. 10:**
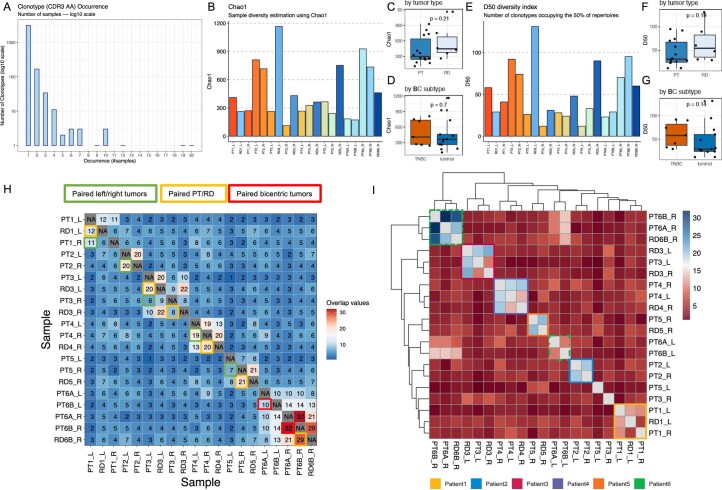
Repartition of the clonal TCR repertoire assessed by TCR Beta-chain CDR3 sequences at the cohort level. The analyses are performed in the in left, right, pre and post-NAC samples of the cohort of 6 patients (20 samples). The clonotypes are identified with the MixCR algorithm^37^ and compared using Immunarch;^38^ A. Repartition of the clonotypes in the cohort; B: TCR Diversity using Chao-1 estimator of species richness; C. by tumor type (PT *versus* RD); D. by BC subtype; E: D50 Diversity index (number of clonotypes occupying the 50% of the repertoires); F. by tumor type (PT *versus* RD); G. by BC subtype; H. Total number of shared clones between samples against “public” clonotypes; I. with clustering. The colored bordered squares represent the comparison of paired data (samples of the same side of a patient (red bordered squares), the left and the right sides (green bordered squares), and between PT and related RDs (yellow bordered squared). In all the boxplots (Extended Data Fig. 10C-D, F-G), lower and upper bars represent the first and third quartile, respectively, the medium bar is the median, and whiskers extend to 1.5 times the inter-quartile range. Statistical tests were two-sided Wilcoxon tests (Extended Data Fig. 10C-D, F-G). L, left; NAC, neoadjuvant chemotherapy; PT, primary tumor; R, right; RD, residual disease; T-cell receptor (TCR).

To further investigate the T cell response to NAC and to compare infiltrating T cell receptor (TCR) repertoires across patients, we extracted TCR beta CDR3 sequences from the RNA-seq data using MixCR^31^ and immunarch^32^. The large majority of clonotypes retrieved were unique to a sample (Extended Data Fig. 10a), but some sequences were found in multiple samples. The proportion of samples annotated in VDJdb, a curated database of TCR sequences of known antigen specificity^33^, was low (1%) and was not different in sequences unique to a sample and in sequences shared within the cohort (8/638 versus 31/3,126, respectively, *P* = 0.7). We evaluated the diversity of the TCR repertoires using the Chao-1 estimator of species richness (Extended Data Fig. 10b–d) and the D50 diversity index (Extended Data Fig. 10e–g), and they were not different by breast cancer subtype nor PT or RD character of the sample. To measure repertoire similarity, we calculated the total number of shared clones between samples against ‘public’ clonotypes (Extended Data Fig. 10h). We found shared TCRs between individuals at a low frequency, whereas most common sharing relationships were found between PT and RD (yellow-bordered squares) and, to a smaller extent, between left and right tumors (green-bordered squares), although the median number of shared clonotypes was not statistically significant (20 versus 11, *P* = 0.12). Except for two samples that showed low sharing with any other sample (PT3_R and PT5_L), clonotypes of the same patients consistently clustered together, either with the contralateral side or with the corresponding RD/PT, consistent with a systemic effect of TILs (Extended Data Fig. 10i).

## Discussion

In the current study, we conducted a large comprehensive overview of sBBC, integrating clinical and pathological data with immune infiltration and genomic profiles generated using modern WES and RNA-seq technologies. Our work thus provides important insights to understand the relationships among tumor, host, immunity and response to treatment.

First, sBBCs represent two distinct and independent diseases occurring incidentally at the same time. In line with previous studies^34–37^, we found a high concordance between the clinical and biological patterns within pairs of sBBCs. Here we demonstrate clearly that these tumors were genomically independent in terms of mutations, CNAs, expression patterns and clonal composition. This finding is in line with most published studies^9,12,15,18^. We also identified genomically related profiles in multicentric tumors, thus confirming that multi-focal tumors represent intra-mammary dissemination of a single breast cancer^8,12,13,38^. These results suggest that the occurrence of sBBCs is explained by non-genetic factors^39^, although very little data are currently available on the link between environmental factors and sBBCs.

Second, we found that the immune infiltration was not determined purely by local tumor microenvironment properties but was different according to the subtype of the contralateral tumor. Several hypotheses can be drawn to explain this observation. First, the immune system might be activated by an index tumor, and immune cells activated by this process might spread to the contralateral tumor. Second, as luminal breast cancers associated with a contralateral tumor of another subtype were associated with a lower degree of ‘luminalness’ (estrogen receptor (ER) and progesterone receptor (PR) staining), we cannot exclude that the highest immune infiltration is derived from such patterns rather than from the presence of the contralateral tumor.

Third, in luminal breast cancers, response to NAC was significantly higher in the case of discordant subtypes of contralateral tumor than in concordant pairs, as with TIL levels. Evidence regarding the influence of contralateral tumor on the response to treatment has not been described so far. Reinisch et al.^40^ previously reported that patients with BBCs had lower pCR rates than patients with unilateral breast cancer (12.6% versus 20.9%). After reanalyzing this dataset^41–44^, we validated independently the higher response rates in patients with discordant luminal breast cancers than in concordant cases, and this finding was also reproducible in a third validation cohort from the SEER program. Hypotheses explaining the difference of the rates of pCR according to the contralateral tumor could be the different baseline immune infiltration levels leading to an increased efficacy of the chemotherapy as previously described^24^ and/or changes in the chemosensitivity of an index tumor by the presence per se of a contralateral tumor. In addition, several other factors, such as different NAT regimen or the time length of treatment, could have modified response rates to NAT.

Finally, the TCR analyses identified that patient was the main source of variability of TCR, and TCRs were not differentially shared between pairs of left and right tumor than they were between pairs of PT–RDs. However, we cannot exclude that some sequences of TCR could have been missed due to the unspecificity of the whole-transcriptome approach against a specific CDR3 approach and due to the bulk tumor transcriptome analysis versus the identification of TCR repertoire specifically on TIL subsets. At a time where bilateral tumor contexts represent a model of growing interest to understand mechanisms underlying immune response to anti-cancer treatment in mice^37,45^, we provide human data regarding the temporal analysis of the TCR repertoire in sBBCs.

Our study has several strengths, such as the use of modern technologies. WES is more informative than targeted sequencing for determining clonal relationships. Second, we studied a very rare and unique cohort of patients, enabling direct comparison of left versus right PT together with a temporal analysis comparing paired samples before versus after NAC. Beyond the challenges in analyzing tumor evolution from bulk sequencing data, we were able to leverage multiple tumor samples to reconstruct a clonal phylogeny from germline data to left and right sBBCs both before and after treatment. Third, our data on immune infiltration are novel contributions to the literature and provide insights into the immune mechanisms underlying the biology of sBBCs.

This study also has limitations. We were able to sequence only a limited number of cases, and a subset of clonally related sBBCs could possibly be identified if larger cohorts were sequenced. Second, the cohort of patients with multiomics data was enriched in patients with *BRCA* mutations, and the latter might represent tumors with particular biological patterns. Third, characterization of the immune microenvironment by bulk sequencing approaches has inherent limitations and is hampered by the absence of ‘ground truth’ data. New insights could be generated by in situ single-cell technologies or through specific transcriptome of the CDR3 region or the TIL subset for analyzing TCR repertoire. Similarly, measurement error in the assays—notably to determine the subtype of breast cancer—could have modified some of our results, although the estimation of the proportion of errors was deemed not exceeding 2%. Finally, no formal causality can be inferred from human observational data, even though the findings of our studies were reproducible in independent validation cohorts.

To conclude, our data suggest that the similarity of molecular portraits in sBBCs could be influenced by common environmental factors and do not support the evidence of a common genetic clonal alteration. Both tumor immune infiltration and response to treatment are differentially associated with the subtype of breast cancer according to the concordant or discordant character of the contralateral tumor. Pairs of tumors from different subtypes of breast cancers should be considered as singular entities before primary systemic treatment is considered, as observed responses might deviate from well-known profiles of response to chemotherapy.

## Methods

The research complies with all relevant ethical regulations and was approved by the institutional review board of Institut Curie (Breast Group) on 6 July 2016.

### Patients and treatments

We identified a cohort of 17,575 female patients with non-metastatic breast cancer treated at the Institut Curie (Paris and Saint-Cloud, France) between 2005 and 2015 in the institutional database (CNIL no. 1766392-v1; data collection and storage in REDCap 12.4.14 and MACRO version 3). Patients were treated according to local guidelines. When indicated, chemotherapy was administered in a neoadjuvant or adjuvant setting; endocrine therapy was indicated in the case of positivity for hormone receptor and according to prognostic factors; and patients with *HER2*^*+*^ tumors received neoadjuvant and/or adjuvant trastuzumab from 2007 onwards. This study was approved by the Breast Cancer Study Group and by the institutional review board of Institut Curie and was conducted in accordance with institutional and ethical rules regarding research on tissue specimens and patients. sBBCs were defined as the occurrence of primary tumors occurring in both breasts with a time interval no greater than 6 months. Metachronous breast cancers, defined as a time interval greater than 6 months between the diagnoses of the first and second tumors, were not included in the current study. Patients with exclusive ductal carcinoma in situ (DCIS) in one of the two sides were excluded from the analyses. Written informed consent was obtained for all patients included in the genomic analyses. No participant received any compensation. In the cohort of patients with sBBCs treated with NAT, regimens were as follows: neoadjuvant chemotherapy (*n* = 50, 46 of whom received anthracyclines/taxanes-based sequential regimen) or neoadjuvant endocrine therapy (NET, *n* = 20, 18 of whom received aromatase inhibitors).

### Tumor samples, subtype of breast cancer and pathological review

In accordance with guidelines used in France^46^, cases were considered ER or PR positive if at least 10% of the tumor cells expressed ERs and/or PRs. *HER2* expression was determined by immunohistochemistry with scoring in accordance with American Society of Clinical Oncology (ASCO)/College of American Pathologists (CAP) guidelines^47^. Scores 3+ were reported as positive; scores 1+/0 were reported as negative (−). Tumors with scores 2+ were further tested by fluorescence in situ hybridization (FISH). *HER2* gene amplification was defined in accordance with ASCO/CAP guidelines.

The subtype of breast cancers were defined as follows: tumors positive for either ER or PR and negative for *HER2* were classified as luminal; tumors positive for *HER2* were considered *HER2*^+^ breast cancer; tumors negative for ER, PR and *HER2* were considered to be TNBCs. In case of multiple tumors in the same breast, the subtype classification was made based upon the tumor of the largest diameter.

Bulk tumor specimens—and the corresponding pretreatment core needle biopsy specimens in case of neoadjuvant treatment—were reviewed by an expert in breast pathology (M.L.). All tumoral tissues studied were formalin-fixed, paraffin-embedded (FFPE) samples.

In the cases with bilateral invasive carcinoma, TILs were reviewed specifically for the purposes of this study, between September 2016 and March 2017. In accordance with the recommendations of the international TILs Working Group^25^, we checked for presence of a mononuclear cell infiltrate in the stroma on H&E-stained sections without additional staining, after excluding areas around DCIS, and tumor zones with necrosis and artifacts. Infiltrates were scored on a continuous scale, as the mean percentage of the stromal area occupied by mononuclear cells. We also assessed intratumoral TIL levels and TIL levels after NAT. Intratumoral TILs were defined as intraepithelial mononuclear cells within tumor nests or in direct contact with tumor cells, and stromal TILs were defined as mononuclear inflammatory cells within intratumoral stromal area and were reported as percentage of stromal area. After NAT, we assessed TIL levels within the borders of the residual tumor bed, as defined by the residual cancer burden (RCB)^48^ and as recommended by the TILs Working Group^26^. To ensure that the mononuclear cells infiltrate considered as TILs in the analyses indeed corresponded to lymphocytes, we carried out CD3^+^ immunostaining on a subset of 24 specimens, which strongly correlated with the levels of unstained TILs (Supplementary Fig. 9). We defined pCR as the absence of invasive residual tumor from both the breast and axillary nodes (ypT0/is N0).

### Concordance between the tumors of sBBC pairs

We evaluated the concordance of the clinical and pathological characteristics between the two tumors within the same patient. Pairs of sBBCs composed of tumors of the same subtype of breast cancers were classified as concordant and, where otherwise, classified as discordant. As breast cancer subtype is known to be the main determinant of tumor biology (notably tumor grade and proliferation), immune infiltration, response to anti-cancer agents and, ultimately, oncologic outcomes, we displayed the results of the clinical section of the paper according to the concordance.

### Independent datasets used in this study

To validate our findings, we analyzed different types of data from three validation cohorts:

- Validation cohort 1 was a breast cancer dataset from the SEER program of the National Cancer Institute, which collects data on cancer diagnoses, treatment and survival for 35% of the US population. Based on 396,179 SEER medical records of patients with breast cancer diagnosed in the 2010–2016 period, we identified 8,367 patients with sBBCs (*n* = 16,734 tumors), defined as two breast cancers diagnosed within 6 months of the primary index diagnosis. This cohort was used to describe the concordance of the breast cancer subtype of the pairs of SBBCs and their relative repartition. To assess response to NAC, we used a subcohort of patients treated with NAC (*n* = 467 patients, representing 934 tumors). We calculated the axillar response rate to NAC, defined as the proportion of patients with a post-NAC number of positive nodes >1 divided by the total number of patients. No data were available on tumor immune infiltration. The use of the SEER database as a validation cohort was approved by the institutional review board of Institut Curie, and access to data followed the standard request access to SEER data.
- Validation cohort 2 was obtained from the GBG and was composed of 105 patients with sBBCs treated within four NAC published trials (GeparTrio^41^ (NCT00544765), GeparQuattro^42^ (NCT00288002), GeparQuinto^43^ (NCT00567554) and GeparSixto^44^ (NCT01426880) trials). Validation cohort 2 was used to validate data on immune infiltration and response to NAT. The GeparTrio, GeparQuattro, GeparQuinto and GeparSixto trials were approved by ethics committees, and all patients consented to the reuse of their data.
- Validation cohort 3 was an in-house cohort composed of patients diagnosed with sBBC at the Institut Curie/René Hugunin between 1984 and 1998 and with bilateral frozen material on bulk tumor specimens of sufficient quality to perform whole-exome analysis. Eight patients with sBBCs had left and right tumor sequencing, but both left and right samples were of sufficient quality for seven patients only. This retrospective study on human biological samples and clinical data collected during care was validated by the Institut Curie institutional review board on 5 July 2021 under reference DATA210188. As per French Public Health Law (art L 1121-1-1 and art L 1121-1-2), written consent is not required for human non-interventional and retrospective studies.

### Sample preparation and next-generation sequencing analyses

DNA and RNA were obtained from the Biological Resource Center of Institut Curie. After selecting patients treated with NAC, tumors from which sufficient frozen material from both left and right tumors was available in the institutional tissue bank both before treatment (defined as PT) and after treatment (in case of RD) were included. Fresh-frozen samples were subjected to genomic DNA extraction and DNA qualification using the QuBit system.

#### DNA pre-processing

One microgram of genomic DNA from each sample was subjected to shearing using the Covaris system, and Illumina-compatible libraries were performed according to Agilent SureSelect XT2 library protocol consisting in repairing DNA ends and ligating Illumina barcoded adapters, followed by polymerase chain reaction (PCR) amplification. Libraries were pooled in equimolar condition before being hybridized on dedicated biotinylated RNA probes targeting whole-exome sequences (Agilent Human All Exon V5 capture probes). After selection using streptavidin beads and PCR amplification, enriched library pools were subjected to qPCR quantification using the KAPA Library Quantification Kit (Roche). Sequencing was carried out on the HiSeq 2000 instrument from Illumina based on a 2 × 100 cycles mode (paired-end reads, 100 bases) using high-output flow cells to produce over 50 million and 170 million paired-end reads for 30× (germline) and 100× (tumors), respectively.

#### DNA sequencing

Samples were sequenced to a median depth of coverage of 153 reads, with 95% of exonic bases passing 50× coverage. Reads were aligned on the human genome reference hg19/GRCh37 by Burrows–Wheeler Aligner^49^ version 0.7.5a; filtering of reads was based on target intersection, mapping quality and PCR duplicate removal, using Picard^50^, BEDTools^51^ and SAMtools^52^, and pre-process using GATK^53^ for local realignment around indels and base score recalibration. Preliminary variant calling was performed using Mutect2 (ref. ^54^) for tumor samples and haplotype caller^55^ for normal samples. Germline mutations are reported in Supplementary Table 12. SuperFreq version 1.3.2 (ref. ^56^) performed annotation and filtering of somatic indels and single-nucleotide variants (SNVs), copy number and purity estimation and subclonal reconstruction, using SNVs, indels and CNAs. SuperFreq was run to analyze together the samples of the same side for each patient. We performed an additional filtering of alterations present in either dbSNP^57^ or ExAC^58^ at a frequency greater than 0.2 or after manual review of the alignment on the Integrative Genomics Viewer or a Somatic Score computed by SuperFreq greater than 0.5. Two samples (RD4_R and RD6B_R) were discarded from the analyses owing to insufficient quality criterion (purity <0.3 and relative difference between the number of mutations in the PT and in the RD superior to 25%).

We computed the pairwise cosine similarity among the copy number calls at the gene level. To ensure a relevant computation, we subtracted the reference copy number (that is, 2) from the calls. The metric then reflects losses and gains, with some tolerance to the exact copy number. We used the function ‘cosine_similarity’ from the module ‘sklearn.metrics.pairwise’ in the package scikit-learn version 0.21.3 (ref. ^59^).

#### Somatic mutations interpretation

Somatic variants were annotated using VEP (version 104)^60^. A variant was denoted as driver if the mutation was present as a splice site, a nonsense, a frameshift or a non-synonymous SNV or indel in COSMIC census gene. The percentage of shared mutations in pairs (pair left–right; pair PT–RD; pair of two multicentric tumors of the same side) was defined as the intersect between the mutations present in both tumors × 2 divided by the sum of the mutation in the two tumors of the pair × 100.

#### Mutational signature deconvolution

The contribution of mutational signatures to individual tumor samples was explored using the signatures deconvoluted by Alexandrov et al.^29^ and referenced in the COSMIC database. We restricted the analyses to the 13 signatures previously evidenced in breast cancer. Signature activities were estimated using the decompTumor2Sig algorithm^61^ in the musicatk (version 1.0.0) R package^62^. The percentage of mutational signatures was calculated by summing the relative contribution of each signature in PT samples to the whole tumor spectrum, divided by the number of sampled, and the result was multiplied by 100. To avoid overrepresentation of patient 6 to the cohort and because signature profiles were similar on each side, we averaged the values of the left side on the one hand and the values of the right side on the other hand.

#### Neoantigens

The VCF files were converted to FASTA format and annotated using the convert2annovar.pl (which converts ‘genotype calling’ format into ANNOVAR format) and annotate_variation.pl (which classifies them as intergenic, intronic, non-synonymous single-nucleotide polymorphism (SNP), frameshift deletion or large-scale duplication) scripts in the ANNOVAR tool. We next used the ANNOVAR coding_change.pl script to infer mutated protein sequence to determine potential premature stop codons. Using these mutated amino acid sequences predicted from annotated non-synonymous variant calls, the peptide sequence surrounding the amino acid corresponding to the new variant was extracted for epitope prediction. As binding of the epitopes to major histocompatibility complex class I (MHC-I) is dependent on the patient-specific HLA alleles, HLA haplotypes were cross-referenced with HLA haplotypes determined by Seq2HLA, before executing netMHCpan for neoantigen prediction, outputting strong and weak binders. Only strong binders were used for neoantigen analysis.

#### RNA pre-processing

Total RNA extracts from tumor samples were subjected to quality control on a Bioanalyzer instrument, and only RNA with RNA integrity number (RIN) >7 were used for sequencing. RNA quantification was achieved using absorbance at 260 nm with a NanoDrop spectrophotometer. RNA-seq libraries were prepared from 1 µg of total RNA using the Illumina TruSeq Stranded mRNA Library Preparation Kit (following the provider’s instructions), which allows performing a strand-specific sequencing. In brief, a first step of polyA+ RNA selection using oligodT magnetic beads is done to focus sequencing on polyadenylated transcripts. After fragmentation, cDNA synthesis was performed, and resulting fragments were used for dA-tailing and then ligated to the TruSeq indexed adapters. PCR amplification is finally achieved to create the final cDNA library. After qPCR quantification using the KAPA Library Quantification Kit (Roche), sequencing was carried out using 2 × 100 cycles (paired-end reads 100 bases).

#### RNA-seq

Sequencing was performed by multiplexing barcoded libraries with the Illumina HiSeq2000 instrument using high-output flow cells to obtain 100 million paired-end reads per sample. Alignments were performed on human reference sequences using TopHat^63^ version 2.0.621. Reads with mapping quality <20 and reads marked as duplicates by Picard version 1.97 were excluded from further analysis. Gene-level read counts were obtained using HTSeq-count^64^ and RefSeq hg19/GRCh37. RNA-seq data are provided as raw counts in Supplementary Table 11.

#### Selection of the genes with the most variant expression, clustering and PCA

We selected the most variant genes, based on the inflection point of the interquartile range (IQR) distribution for gene expression. The gene expression was previously rlog-transformed with DESeq2 (1.22.1)^65^. This method is more data-driven than a fixed threshold to define the proportion of genes with the highest level of variation. For each gene, we applied the following procedure: (1) we calculated the IQR for all samples; (2) we sorted the IQR values of the genes in ascending order, to generate an ordered distribution; (3) we estimated the major inflection point of the IQR curve as the point on the curve farthest away from a line drawn between the start and end points of the distribution; and (4) we retained genes with an IQR higher than the inflection point. Hierarchical clustering was performed with Pearson correlation and Ward linkage. We next performed PCA to reduce dimensionality using the 3,000 most variable genes.

#### CIBERSORT

CIBERSORT^30^ is an analytical tool quantifying the levels of distinct immune cell types within a complex gene expression mixture. We applied the original CIBERSORT gene signature LM22 defining 22 immune cell subtypes to all the samples of the cohort, the number of permutations being set to 100 and the mode being set to ‘absolute’. For each immune subpopulation, (1) we calculated the difference between a given sample and the rest of the cohort; (2) we squared the result; and (3) we summed the difference between this patient and each other sample of the cohort, resulting in a dissimilarity index. We displayed the overall results on a correlogram.

#### Multiplexed immunofluorescence and immunohistochemistry

To characterize the microenvironment of these samples using an orthogonal experimental approach, we performed immunofluorescence stainings using two antibody panels (panel 1: CD8 CD45RO CD20 CD4 and FoxP3; panel 1: CD4 – CD8 – CD45RO – FOXP3 – CD20 – pan-cytokeratin; Extended Data Fig. 7a–f; panel 2: CD68 – CD163 – CD138 – CKIT – pan-cytokeratin; Extended Data Fig. 7g,h) to assess the concordance between the immune subpopulations inferred by gene expression and the number of cells stained on pathologic slides. We performed multiplexed immunofluorescence staining on the 20 samples of the cohort (14 microbiopsies and six residual tumor samples). Immunostaining was processed in a Bond RX automated (Leica) with Opal 7-Color IHC Kits (Akoya Biosciences, NEL821001KT) according to the manufacturer’s instructions. We used two different panels of antibodies: panel 1: CD4 (clone EP204) – CD8 (clone C8/144B) – CD45RO (clone 2B11 + PD7/26) – FOXP3 (clone 236A/E7) – CD20 (clone L26) – pan-cytokeratin (clone AE1/AE3); panel 2: CD68 (clone KP1) – CD163 (clone 10D16) – CD138 (clone MI15) – CKIT (polyclonal) – pan-cytokeratin (clone AE1/AE3). After applying DAPI for visualization of nuclei, slides were mounted and cover-slipped. Multiplexed slides were scanned using the Vectra 3 automated quantitative pathology imaging system (Vectra 3.0.5, Akoya Biosciences), and images were analyzed using inForm Advanced Image Analysis Software (inForm 2.6.0, Akoya Biosciences). We used the total number of stained cells for an antibody to perform the correlation with immune subsets inferred by the deconvolution, with the following correspondence: CD8^+^ cells, cytotoxic T cells; CD4^+^/FOXP3^+^ cells, T regs; CD45RO^+^ cells, T cells memory activated; CD20^+^ cells, B cells naive; CD68^+^/CD163^+^ cells, macrophage M2; CD69+ cells, macrophages M0, M1 and M2; CD138^+^ cells, plasma cells.

#### TCR sequencing analysis

We applied the MixCR algorithm on RNA-seq data of 20 samples to identify and quantify TCR beta chain CDR3 sequences. MiXCR (version 2.1.5)^31^ was used with its default parameters to extract and quantify TCR beta chain CDR3 sequences from RNA-seq FASTQ files. From the MiXCR output, we obtained for each sample the total number of distinct TCR beta clones and the number of reads supporting each clone, and we normalized the result by the total number of reads. We used immunarch^32^ to calculate quantitative descriptors of both the diversity and sharing of the TCR beta chain repertoire. For the estimation of repertoire diversity, we calculated Chao-1, a non-parametric asymptotic estimator of species richness and the D50 diversity index, representing the number of clonotypes occupying the 50% of repertoires. For repertoire similarity, we calculated the total number of shared clones between samples against ‘public’ clonotypes.

### Statistical analysis

The study population was described in terms of frequencies for qualitative variables or medians and associated ranges for quantitative variables. Chi-squared or Fisher tests were performed to search for differences between subgroups for each variable (considered significant for *P* ≤ 0.05). Continuous variables were compared between groups in Wilcoxon–Mann–Whitney tests for groups of fewer than 30 patients and for variables following multimodal distributions. Student *t*-tests were used in all other cases. Pre-NAC and post-NAC TIL levels were analyzed as continuous variables. In case of categorical variables, the kappa coefficient^60^ was computed as a measure of concordance between the left and right side of a same patient (varying from −1 as absolute discordance to + 1 as absolute concordance and 0 as absence of concordance); otherwise, in case of numeric or integer variables, the Kendall test was used. Correlations between continuous variables were calculated using Spearman coefficient. Factors predictive of pCR were introduced into a univariate logistic regression model. A multivariate logistic model with a forward stepwise selection procedure was then applied; the covariates included having a likelihood ratio test *P* ≤ 0.05. The same approach was used for univariate and multivariate linear regression models.

All *P* values no greater than 0.05 were considered significant. In the case where we tested the hypothesis of potentially different effects of the concordant or discordant status of the tumor pair regarding the subtype of breast cancer on immune infiltration or response to treatment, we included an interaction term in a linear regression model or logistic regression model, respectively. A *P* value of 0.10 was selected to determine the statistical significance of the interaction term, as it has been suggested due to a low power of the test in the interaction setting. When appliable, all statistical tests were two-sided.

RFS was defined as the period from surgery to death, locoregional recurrence or distant recurrence, whichever came first. Patients who did not have any of these occurrences documented were censored at the last known contact date. The Cox proportional hazards model was used to determine hazard ratios associated with RFS and associated 95% confidence intervals. In the univariate analysis, variables with a *P* value for the likelihood ratio test of 0.05 or below were chosen for inclusion in the multivariate analysis. The final multivariate model was built using a forward stepwise selection approach with a significance criterion of 5%. In box plots, lower and upper bars represent the first and third quartiles, respectively; the medium bar is the median; and whiskers extend to 1.5 times the IQR. Data were processed and statistical analyses were carried out with R software version 4.2.1 (www.cran.r-project.org).

### Reporting summary

Further information on research design is available in the Nature Portfolio Reporting Summary linked to this article.

## Online content

Any methods, additional references, Nature Portfolio reporting summaries, source data, extended data, supplementary information, acknowledgements, peer review information; details of author contributions and competing interests; and statements of data and code availability are available at 10.1038/s41591-023-02216-8.

## Supplementary information

Supplementary Information Supplementary Figs. 1–9 and Supplementary Tables 1–9.

Reporting Summary

Supplementary Table 10 Mutations per sample and per patient in the cohort of the six patients and 20 samples with next-generation sequencing data

Supplementary Table 11 Raw counts for RNA-seq data per sample and per patient in the cohort of the six patients and 20 samples with next-generation sequencing data

Supplementary Table 12 Germline mutations identified in the in the cohort of the six patients and 20 samples with next-generation sequencing data

## Data Availability

The genomic and transcriptomic data generated during the current study are available as pre-processed files at the following link: https://github.com/rt2lab/bc_bilat_neo (folder data/external). Raw sequence data have been deposited at the European Genome-phenome Archive, which is hosted by the European Bioinformatics Institute and the Centre for Genomic Regulation, under accession number EGAS00001006910.

## Code availability

Codes are available at the following URL: https://github.com/rt2lab/bc_bilat_neo.
