# Supplementary material for: Evolution of synchronous female bilateral breast cancers and response to treatment
Source: Nat Med. 2023 Mar 6;29(3):646–55. doi: 10.1038/s41591-023-02216-8 (PMC10033420; doi:10.1038/s41591-023-02216-8)
Supplement: Supplementary file 1 — Supplementary Figs. 1–9 and Supplementary Tables 1–9. [file 41591_2023_2216_MOESM1_ESM.pdf]

nature medicine

Article

https://doi.org/10.1038/s41591-023-02216-8

Evolution of synchronous female bilateral breast cancers and response to treatment

In the format provided by the authors and unedited

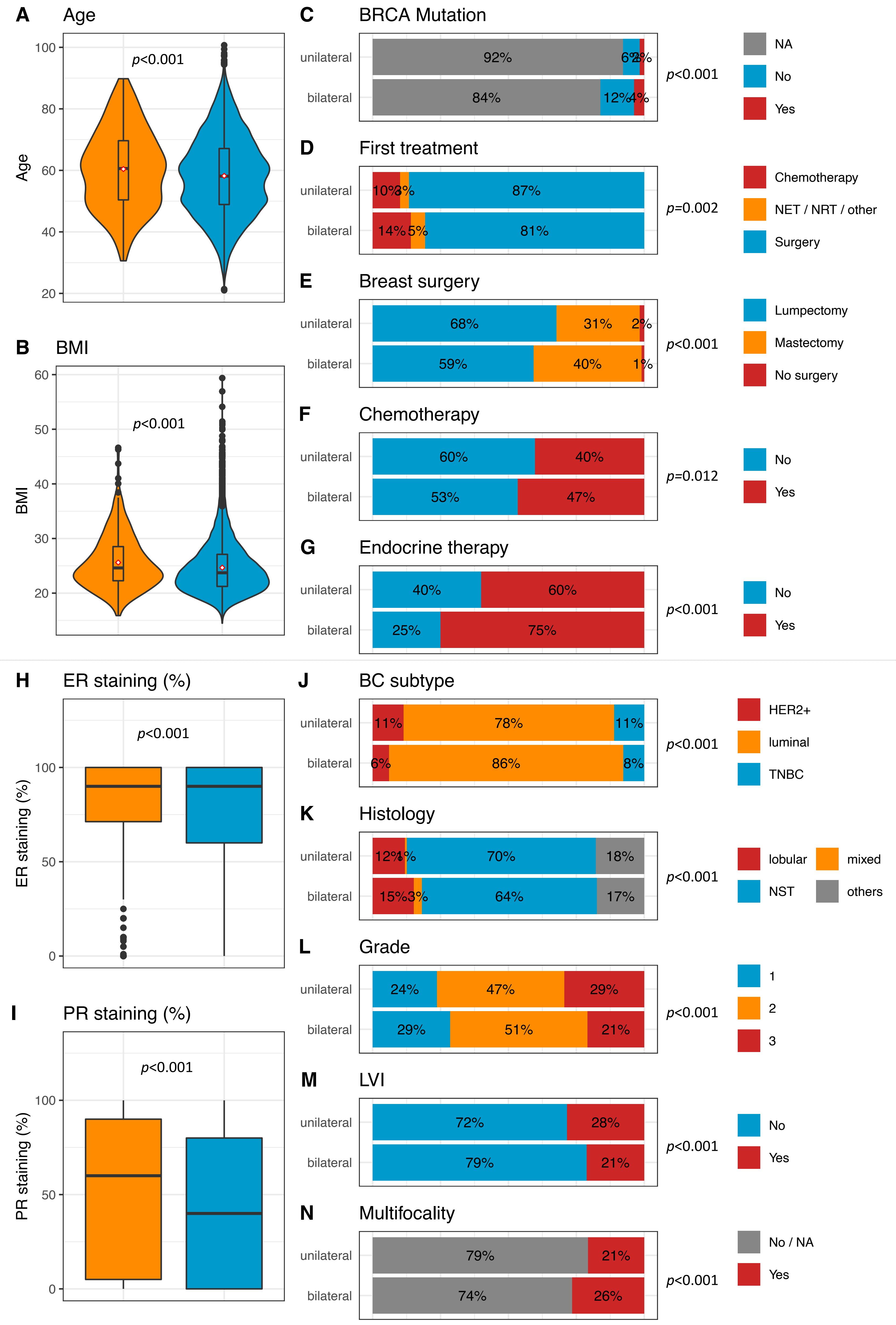

Supplementary figure 1: Comparison of the patient's and tumor's characteristics according to the unilateral or bilateral character of the tumor. The sBBCs cohort comprises 404 patients carrying 804 sBBCs that are compared with 17171 patients with unilateral breast cancers. Comparison of the bilaterality regarding (i) patients characteristics: A. Age at breast cancer diagnosis (n=17173); B. BMI (n=11232); C. Presence of a germline mutation of *BRCA1* or *BRCA2*; D. First treatment; E. Breast surgery; F. Chemotherapy; G. Endocrine therapy; (ii) tumor's characteristics: H. ER staining (as percentage) (n=14500); I. PR staining (as percentage) (n=13755); J. breast cancer subtype; K. Histological type; L. Tumor grade; M. Lymphovascular invasion; N. Multifocality. In all the boxplots (Supplementary figure 1A-B) and in the violin plots (Fig1H-I), lower and upper bars represent the first and third quartile, respectively, the medium bar is the median, and whiskers extend to 1.5 times the inter-quartile range. Statistical tests were chi-square tests (Supplementary figure 1C-G, J-M) and Student t-tests (Fig1A-B, H-I) when indicated. Exact *p*-values for tests in Supplementary figure 1A-C are *p*=0.00057, *p*=0.00019, *p*= 2.08E-05 respectively; Supplementary figure 1E: *p*= 2.00E-07; Supplementary figure 1G-N: *p*= 1.63E-09, *p*=5.1E10-7, *p*= 0.00012, *p*= 3.89E-07, *p*= 2.80E-15, p= 4.13E-06, *p*= 0.0003, *p*= 8.29E-05 respectively. Abbreviations: BMI, body mass index; ER, estrogen receptor; LVI, lymphovascular invasion; NET, neoadjuvant endocrine therapy; NRT, neoadjuvant radiotherapy; PR, progesterone receptor.

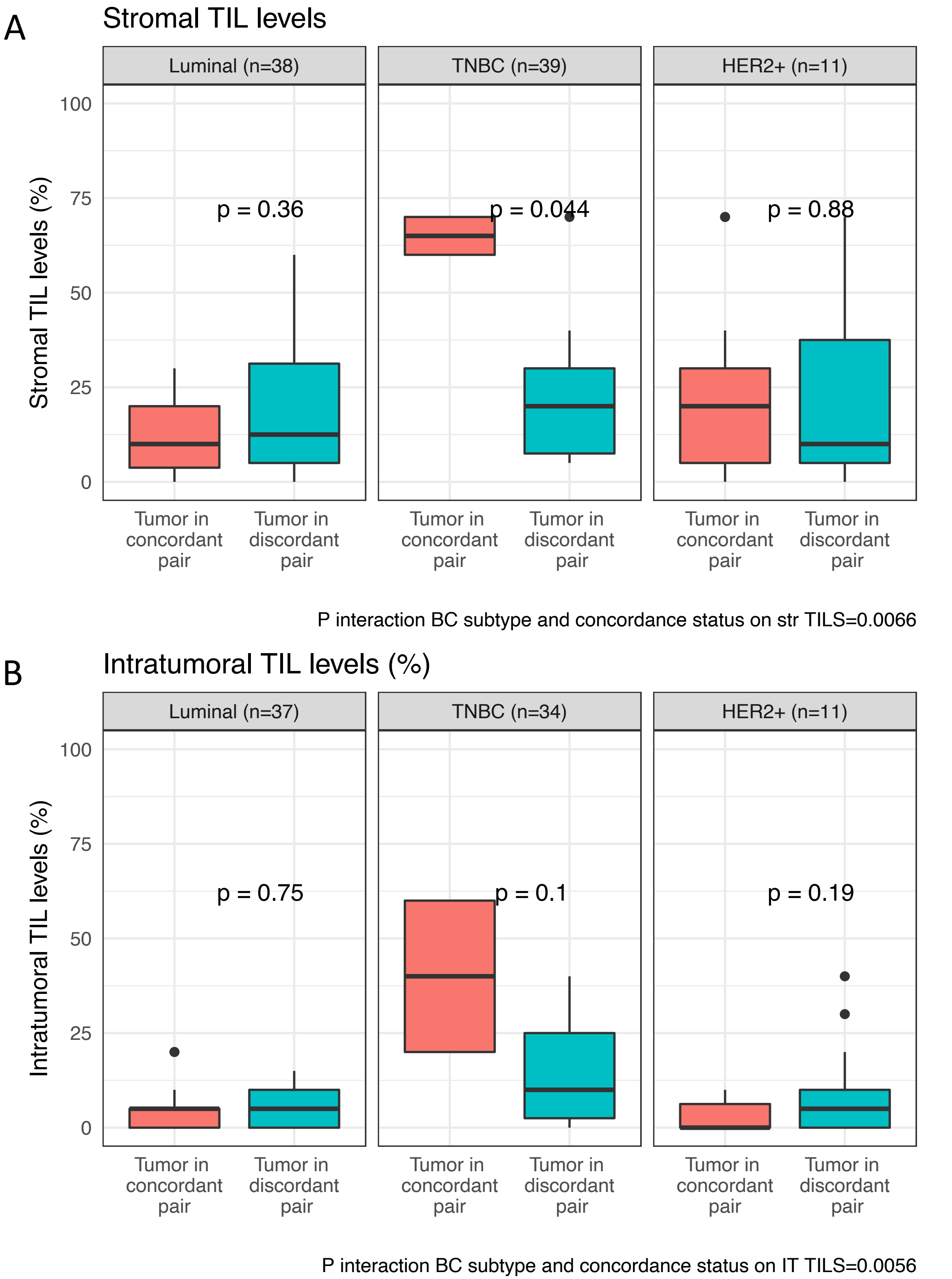

Supplementary figure 2: Immune infiltration levels by breast cancer subtype and by the concordance status of tumor pairs in the validation cohort of the GBG

The cohort from the GBG (German breast group) comprised 105 patients with sBBCs treated within four neoadjuvant chemotherapy trials. A. Stromal TIL levels of the index tumor by breast cancer subtype and by the concordance status of the pair it belongs to; B. Intratumoral TIL levels of the index tumor by breast cancer subtype and by the concordance status of the pair it belongs to. In boxplots, lower and upper bars represent the first and third quartile, respectively, the medium bar is the median, and whiskers extend to 1.5 times the inter-quartile range. Statistical tests were Wilcoxon tests, all tests are two sided.

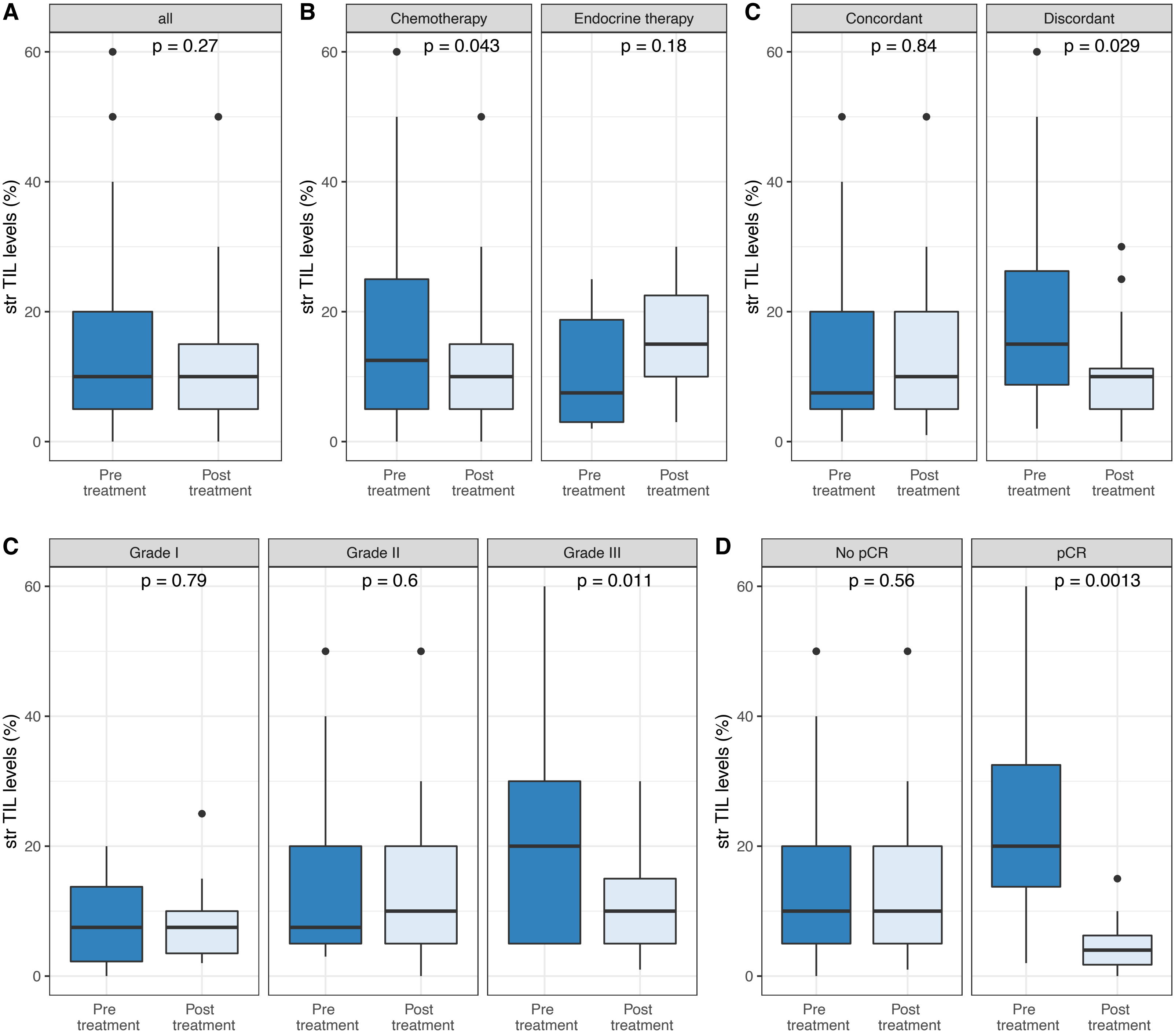

Supplementary figure 3: Stromal TIL levels before and after neoadjuvant treatment (NAT).
The cohort comprises 37 patients (n=74 tumors) with sBBCs treated with neoadjuvant treatment; A. Box plots of pre and post treatment stromal TIL levels in the whole population ; B. according to the type of treatment ; C. according to the concordant or discordant status of the pair; C. according to the tumor grade; D. according to the pCR status. Each boxplot represents the median value and associated interquartile range. In boxplots, lower and upper bars represent the first and third quartile, respectively, the medium bar is the median, and whiskers extend to 1.5 times the inter-quartile range. Statistical tests were Wilcoxon tests, all statistical tests are two sided. Abbreviations: pCR: pathological complete response.

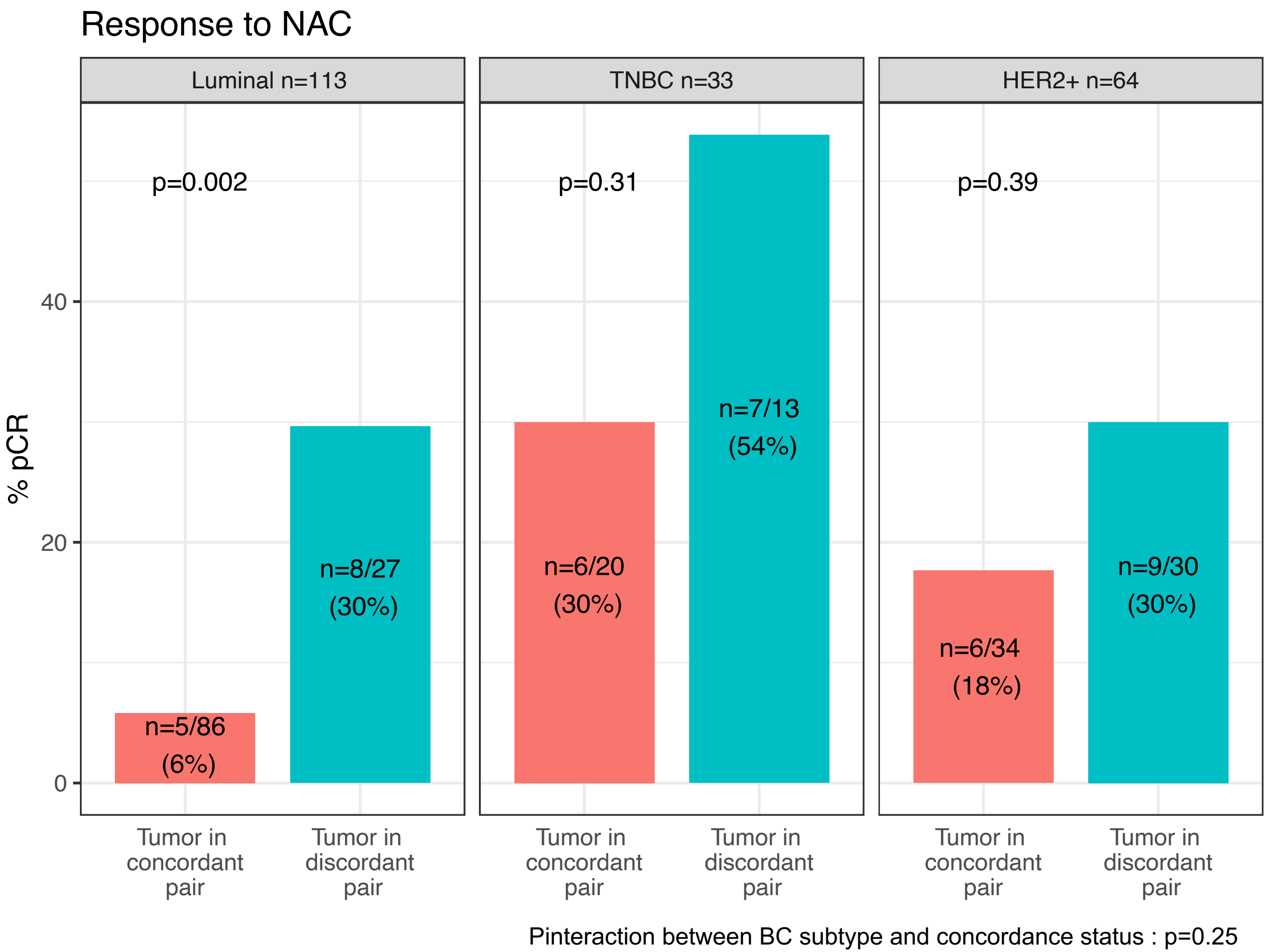

Supplementary figure 4: pCR rates of pairs of sBBCs according to the breast cancer subtype and the concordance status of the tumor pair in the validation cohort of the GBG

The cohort from the GBG (German breast group) comprised 105 patients with sBBCs treated within four neoadjuvant chemotherapy trials. Statistical tests were chi-square tests, all statistical tests are two-sided. Abbreviations: pCR: pathological complete response; sBBCs: synchronous bilateral breast cancer.

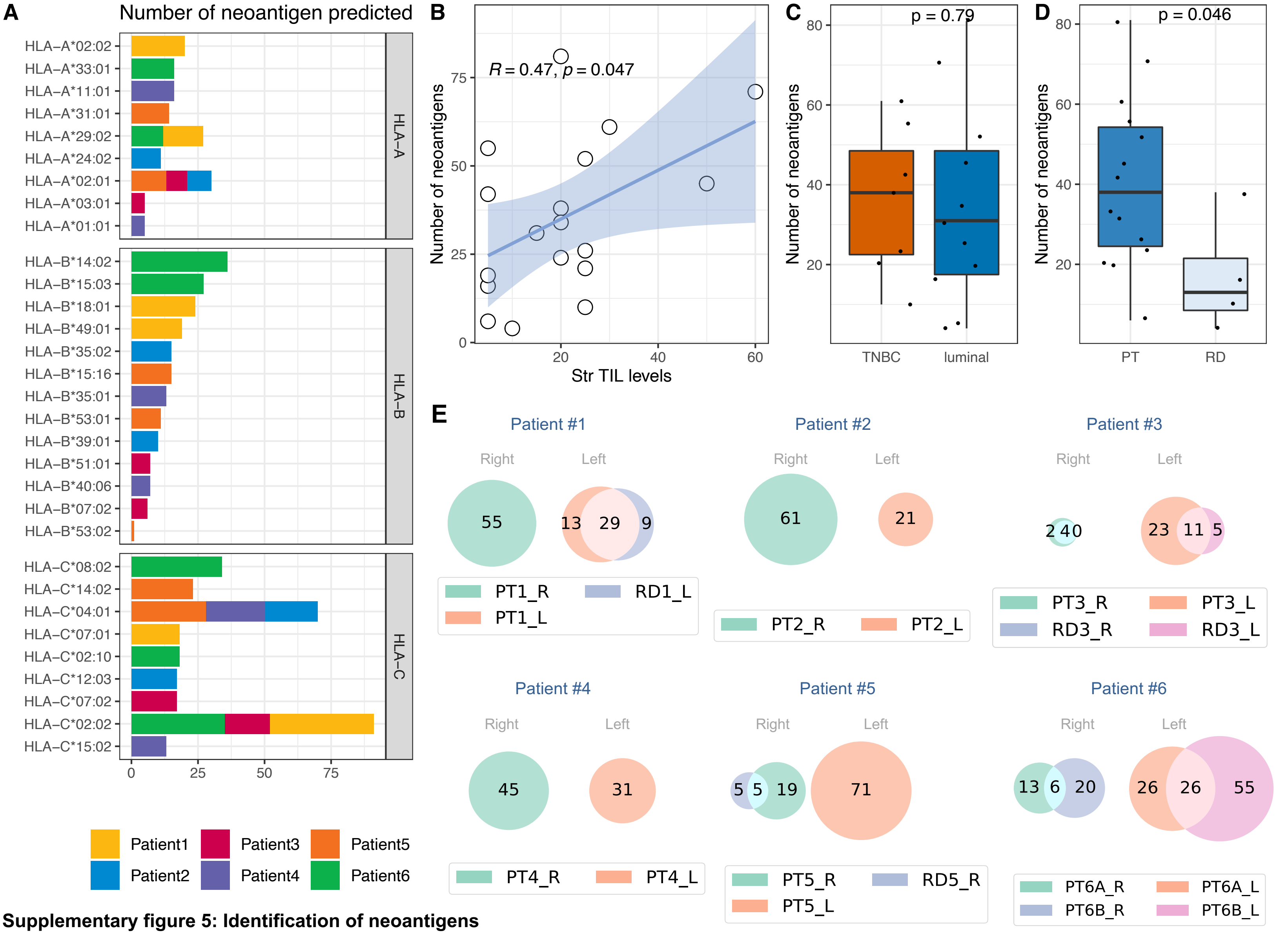

Supplementary figure 5: Identification of neoantigens

The analyses are performed in the in left, right, pre and post-NAC samples of a cohort of 6 patients (20 samples). Number of neoantigens predicted by A. Type of HLA; B. Stromal TIL levels; C. BC subtype; D. Type of sample (PT/RD); E. Neoantigen sharing between samples. Abbreviations: PT, patient; RD, residual disease; str TIL, stromal tumor infiltrating lymphocytes; TNBC: triple negative breast cancer. In all boxplots (Supplementary figure 5C-D), lower and upper bars represent the first and third quartile, respectively, the medium bar is the median, and whiskers extend to 1.5 times the inter-quartile range. Statistical tests were Wilcoxon tests in boxplots and are two-sided and Pearson correlation coefficients in Supplementary figure 5B. The lines represent the fitted linear regression line and the colored bands represent the 95% confidence interval bands.

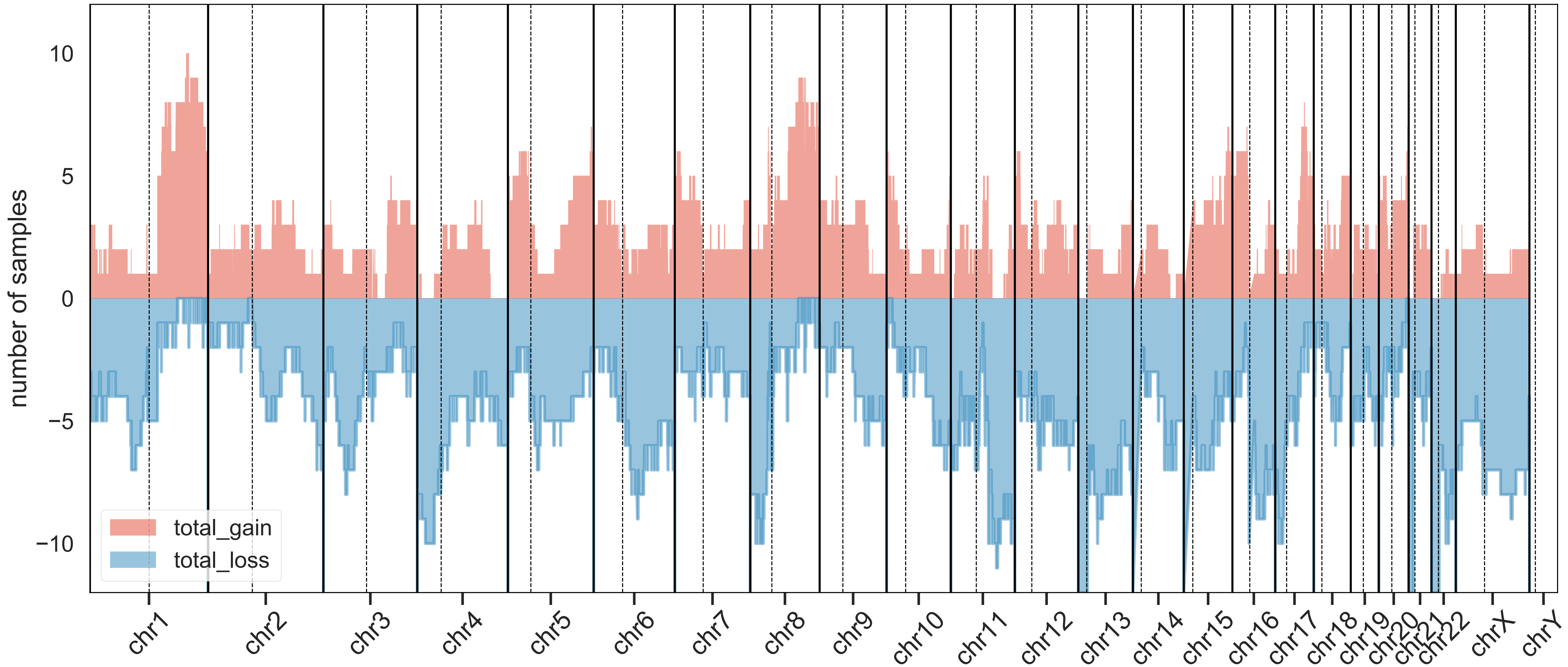

Supplementary figure 6: Copy number patterns of the genomes of the 14 primary tumors.
The total percentage of gain/loss is listed by the cumulative change per sample.
Chr, chromosome

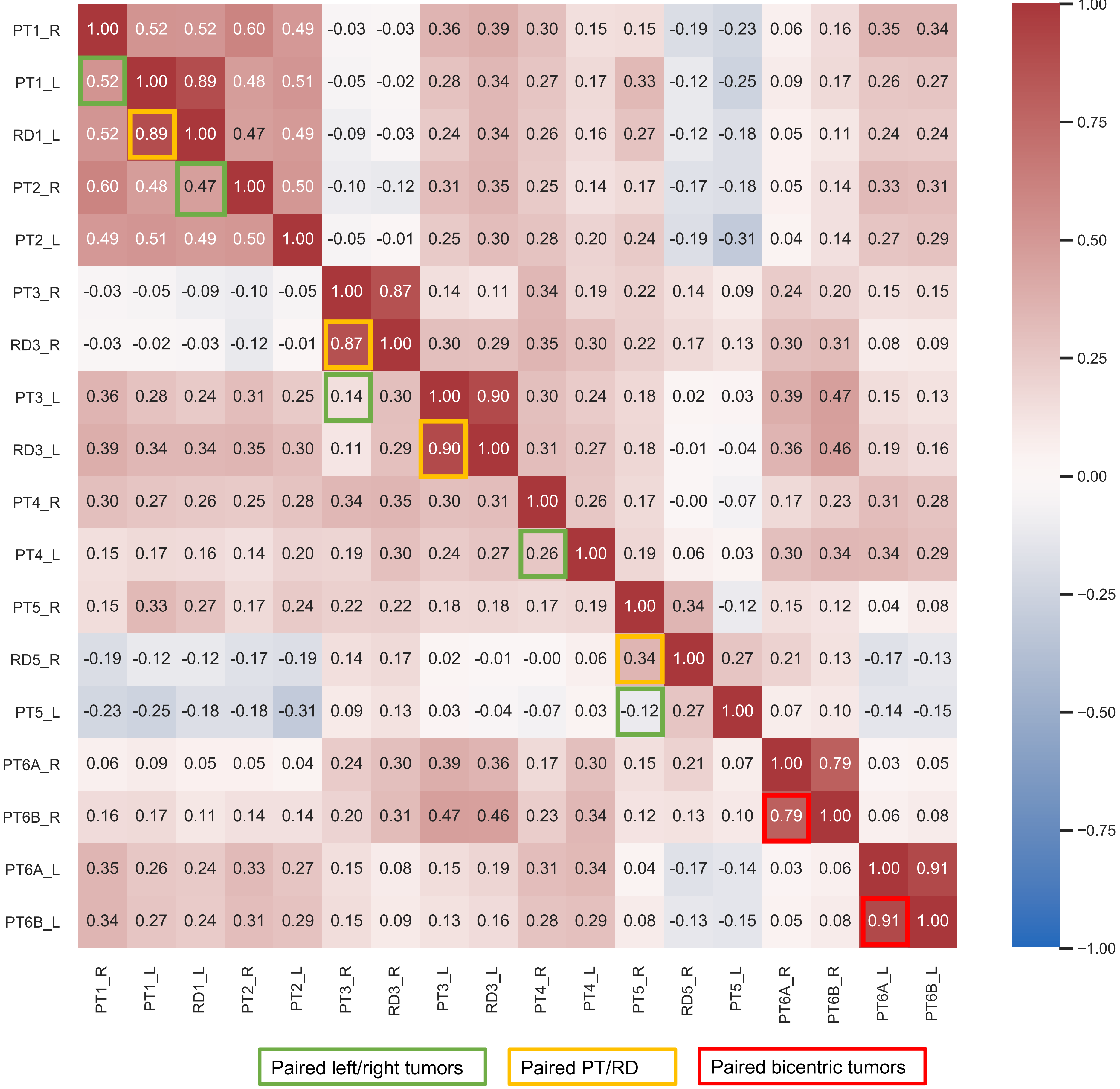

Supplementary figure 7: Similarity between samples regarding copy number profiles.
Pairwise cosine similarity of the copy number profiles of the 18 samples of the cohort with sufficient quality criteria (samples RD4_R and RD6B_R were discarded from the analyses). The colored bordered squares represent the comparison of paired data (left and the right tumors of a pairs of synchronous bilateral breast cancer: green bordered squares; between PT and related RDs: yellow bordered squared; samples of the same side of a patient: red bordered squares.

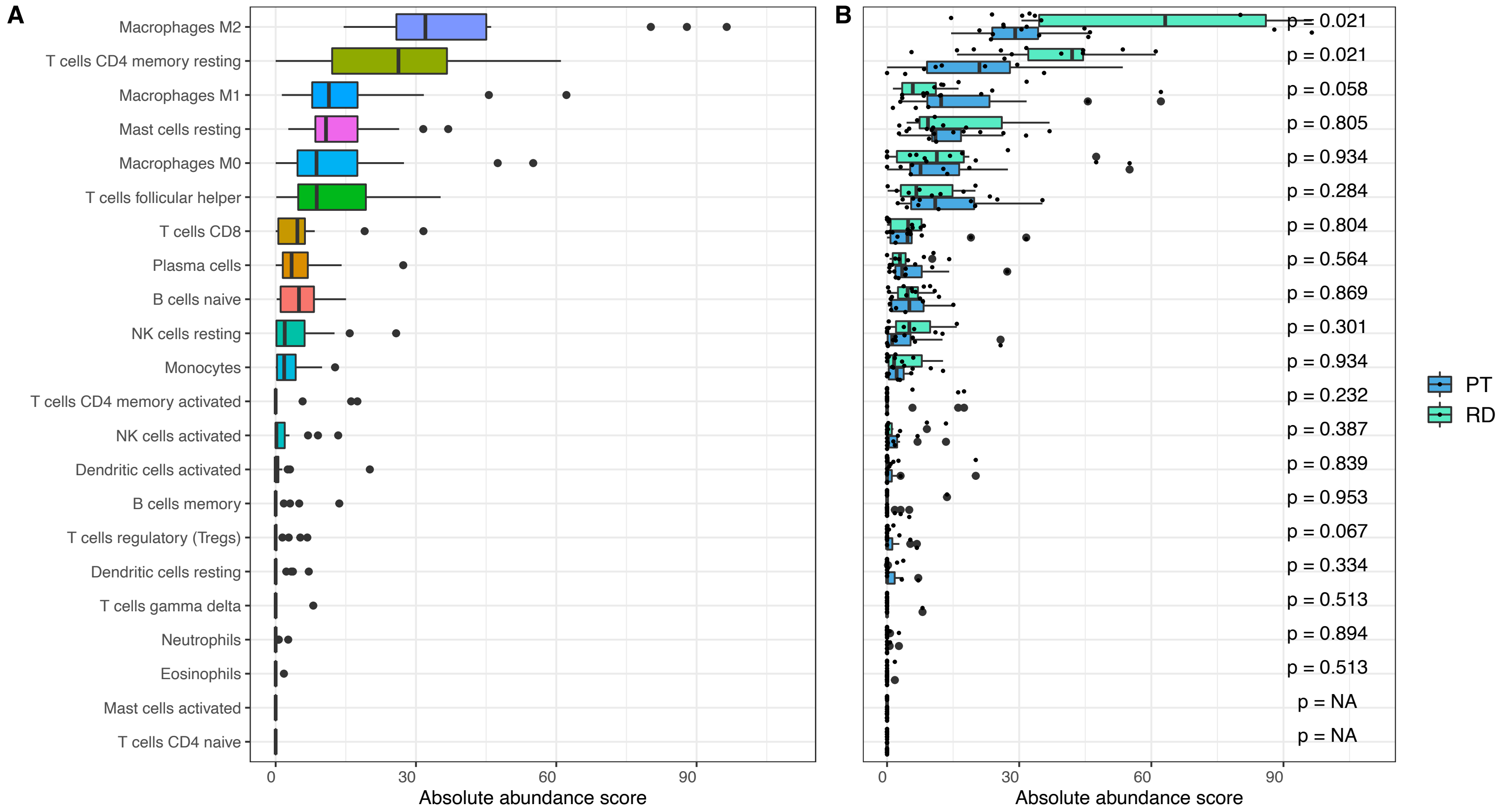

Supplementary figure 8: Composition of the immune microenvironment (22 immune subpopulations) of the sBBCs cohort (from the CIBERSORT algorithm). The analyses are performed in the in left, right, pre and post-NAC samples of a cohort of 6 patients (20 samples). The composition of the immune microenvironment (22 immune subpopulations) was deconvoluted by the CIBERSORT algorithm (absolute mode) as described by Newman; A. Composition of the immune microenvironment in the whole population: B. According to the type of sample (PT *versus* RD) samples;

Abbreviations: L, left; PT, primary tumor; R, right; RD, residual disease; sBBC, synchronous bilateral breast cancer. In boxplots, lower and upper bars represent the first and third quartile, respectively, the medium bar is the median, and whiskers extend to 1.5 times the inter-quartile range. Statistical tests were Wilcoxon tests, all tests are two-sided.

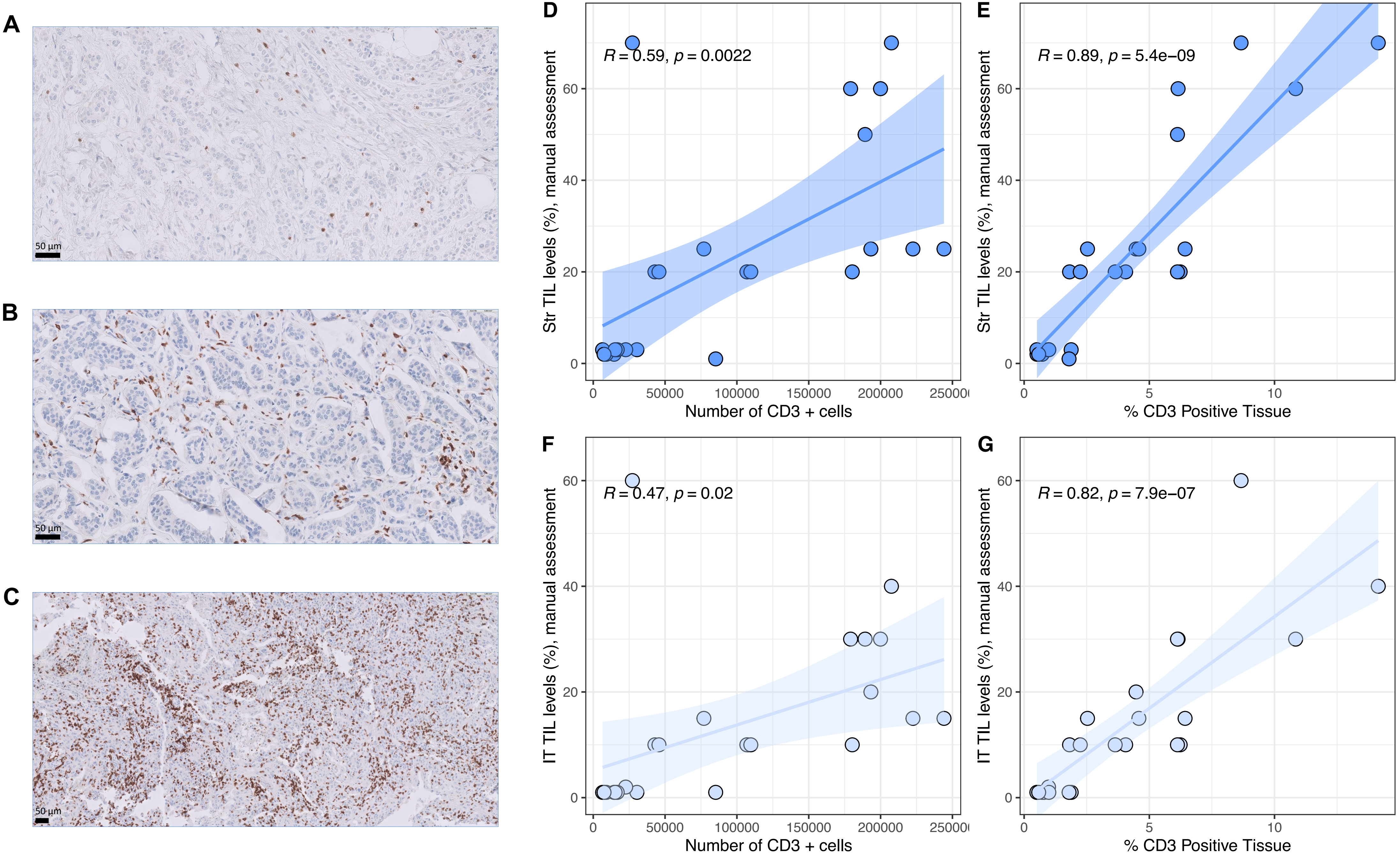

Supplementary figure 9: Comparison between TIL levels on unstained slides and immune infiltration assessed with a CD3 immunostaining.
Immunohistochemistry staining was performed on a subset of 24 samples out of the cohort of 277 samples with TILs manual review available. A: Manual review: stromal TILs 2 %, intratumoral TILs 1%; Percentage of CD3+ tissue: 0.72; B: Manual review: stromal TILs 25 %, intratumoral TILs 15%; Percentage of CD3+ tissue : 4.58 ; C: Manual review: stromal TILs 70 %, intratumoral TILs 40%; Percentage of CD3+ tissue : 14.1; D: Correlation between unstained stromal TILs and the number of CD3+ positive cells; E. Correlation between unstained stromal TILs and the percentage of CD3+ Tissue ; F. Correlation between unstained intratumoral TILs and the number of CD3+ positive cells; G. Correlation between unstained intratumoral TILs and the percentage of CD3 positive tissue. Statistical test was Pearson correlation coefficients. The lines represent the fitted linear regression line and the colored bands represent the 95% confidence interval bands. The staining was performed once for every case. Black scale (bottom left) represents 50 µm.

Supplementary Table 1: Comparison of the patients and tumor characteristics according to the unilateral or synchronous bilateral character of the tumor.

variable	level	bilateral	unilateral	*p*
**Patient level**	**n**	**404**	**17171**	**17175**
**Age**		60.4 (12.8)	58.2 (12.8)	**0.001**
**Age class**	<40	17 (4.2)	1264 (7.4)	**0.01**
	[40-50[	76 (18.9)	3565 (20.8)	
	[50-60[	103 (25.6)	4785 (27.9)	
	[60-70[	110 (27.3)	4323 (25.2)	
	>=70	97 (24.1)	3233 (18.8)	
**BMI**		25.6 (5.0)	24.7 (4.8)	**<0.001**
**BMI class**	<18.5	11 (2.9)	471 (4.2)	**0.019**
	18.5-24.9	193 (51.7)	6441 (57.2)	
	25-29.9	103 (27.6)	2887 (25.6)	
	>=30	66 (17.7)	1460 (13.0)	
**Age at menarche**		12.8 (1.6)	12.9 (1.6)	0.151
**Previous pregnancy**	No	68 (17.9)	1641 (15.0)	0.13
	Yes	311 (82.1)	9316 (85.0)	
**Menopausal status**	Premenopausal	115 (29.9)	4309 (30.0)	1
	Postmenopausal	270 (70.1)	10053 (70.0)	
**Age at menopause**		50.8 (4.6)	50.5 (4.3)	0.249
**Hormone replacement therapy**	HRT	93 (48.4)	2507 (51.2)	0.504
	No HRT	99 (51.6)	2393 (48.8)	
**Familial history breast / ov. cancer**	No	125 (40.6)	3710 (44.2)	0.23
	Yes	183 (59.4)	4682 (55.8)	
**BRCA mutation**	No	51 (12.6)	2381 (13.9)	**<0.001**
	Yes	65 (16.1)	1353 (7.9)	
	NA	288 (71.3)	13437 (78.3)	
**First treatment**	Chemotherapy	57 (14.1)	1739 (10.1)	**0.002**
	NET / NRT / other	21 (5.2)	556 (3.2)	
	Surgery	326 (80.7)	14876 (86.6)	
**Chemotherapy**	No	216 (53.5)	10264 (59.8)	**0.012**
	Yes	188 (46.5)	6907 (40.2)	
**Chemotherapy regimen**	Anthra-taxanes	153 (37.9)	5124 (29.8)	**0.01**
	Anthracyclines	27 (6.7)	1296 (7.5)	
	Others	0 (0.0)	51 (0.3)	
	Taxanes	8 (2.0)	435 (2.5)	
**Endocrine therapy**	No	101 (25.0)	6864 (40.0)	**<0.001**
	Yes	303 (75.0)	10307 (60.0)	
Endocrine therapy type	*Aromatase inhibitor*	*234 (57.9)*	*6761 (39.4)*	**<0.001**
	LH RH agonists	*3 (0.7)*	*71 (0.4)*	
	Others	*0 (0.0)*	*4 (0.0)*	
	Tamoxifen	*66 (16.3)*	*3471 (20.2)*	
**Targeted therapy**	No	374 (92.6)	15635 (91.1)	0.331
	Yes	30 (7.4)	1536 (8.9)	
Targeted therapy type	*Bevacizumab*	*0 (0.0)*	*47 (0.3)*	0.618
	Lapatinib	*0 (0.0)*	*3 (0.0)*	
	Trastuzumab	*30 (7.4)*	*1455 (8.5)*	
**Radiotherapy**	No	78 (19.3)	2335 (13.6)	**0.001**
	Yes	326 (80.7)	14836 (86.4)	

Tumor level	n	808	17382	
**Diagnostic modality**	Non-palpable	498 (64.3)	7149 (61.8)	0.189
	Palpable	277 (35.7)	4415 (38.2)	
**Clinical size**		31.1 (21.0)	27.9 (20.2)	**0.002**
**Clinical T stage**	T0 or pTis	373 (46.6)	4681 (27.7)	**<0.001**
	T1	201 (25.1)	7206 (42.6)	
	T2	169 (21.1)	3838 (22.7)	
	T3	34 (4.2)	779 (4.6)	
	T4	23 (2.9)	414 (2.4)	
**Clinical N stage**	cN0	720 (89.3)	15187 (87.5)	0.139
	cN1/N2/N3	86 (10.7)	2168 (12.5)	
**Pathological T stage**	pT0 or pTis	95 (14.4)	1995 (13.4)	0.519
	pT1	413 (62.6)	9680 (64.8)	
	pT2	136 (20.6)	2986 (20.0)	
	pT3/T4	16 (2.4)	275 (1.8)	
**BC subtype**	luminal	583 (86.2)	11410 (77.5)	**<0.001**
	TNBC	52 (7.7)	1630 (11.1)	
	HER2+	41 (6.1)	1677 (11.4)	
**ER status**	negative	70 (10.2)	2503 (16.8)	**<0.001**
	positive	618 (89.8)	12411 (83.2)	
**PR status**	negative	170 (25.6)	4677 (33.0)	**<0.001**
	positive	494 (74.4)	9500 (67.0)	
***HER2* status**	negative	635 (93.9)	13061 (88.6)	**<0.001**
	positive	41 (6.1)	1677 (11.4)	
**Percentage of ER positivity**		77.8 (31.1)	70.8 (36.3)	**<0.001**
**Percentage of PR positivity**		49.9 (38.7)	43.9 (38.8)	**<0.001**
**Invasive or DCIS**	DCIS only	96 (14.5)	1955 (13.0)	0.269
	Invasive carcinoma	565 (85.5)	13123 (87.0)	
**DCIS component**	DCIS	474 (69.5)	6940 (68.8)	0.726
	No DCIS	208 (30.5)	3150 (31.2)	
**Multifocality**	No / NA	594 (73.5)	13791 (79.3)	**<0.001**
	Yes	214 (26.5)	3591 (20.7)	
**pN status**	0	577 (71.4)	12504 (71.9)	0.736
	[1-3]	162 (20.0)	3525 (20.3)	
	4 and more	69 (8.5)	1353 (7.8)	
**Grade**	1	196 (28.6)	3473 (23.7)	**<0.001**
	2	346 (50.5)	6877 (46.9)	
	3	143 (20.9)	4309 (29.4)	
**LVI**	No	425 (78.8)	8781 (71.6)	**<0.001**
	Yes	114 (21.2)	3482 (28.4)	
**Histological type**	NST	517 (64.4)	12047 (69.6)	**<0.001**
	lobular	122 (15.2)	2068 (12.0)	
	mixed	24 (3.0)	105 (0.6)	
	others	140 (17.4)	3079 (17.8)	
**Breast surgery**	Lumpectomy	479 (59.3)	11767 (67.7)	**<0.001**
	Mastectomy	321 (39.7)	5325 (30.6)	
	No surgery	8 (1.0)	290 (1.7)	
**Axillar surgery**	AND	419 (52.2)	10476 (63.2)	**<0.001**
	SND	303 (37.8)	5039 (30.4)	
	No axillary sampling	80 (10.0)	1068 (6.4)	

Missing data: Age, n=2; Age class, n=2; BMI, n=5943; BMI class, n=5943; BMI class, n=5943; Age at menarche, n=7543; Previous pregnancy, n=6239; Menopausal status, n=2828; Age at menopause, n=9428; Hormone replacement therapy, n=12483; Familial history breast / ov. cancer, n=8875; Research hereditary predisposition, n=13725; BRCA mutation, n=16178; Targeted therapy type, n=31; Diagnostic modality, n=5851; clinical size, n=8481; clinical T stage, n=472; clinical N stage, n=29; pathological T stage, n=2594; breast cancer subtype, n=2797; ER status, n=2588; PR status, n=3349; HER2 status, n=2776; percentage of ER positivity, n=2675; percentage of PR positivity, n=3420; Invasive or DCIS, n=2451; DCIS component, n=7418; histological size, n=5221; grade, n=2846; lymphovascular invasion, n=5388; histological type, n=88; axillar surgery, n=805. Statistical tests were chi-square tests for categorical variables and t-tests or anova tests for quantitative variables, all tests were two-sided. Exact p-values for BMI, BRCA mutation, Endocrine therapy, Endocrine therapy type, Clinical T stage, BC subtype, ER status, PR status, HER2 status, Percentage of ER positivity, Percentage of PR positivity, Multifocality, Grade, LVI, Histological type, Breast surgery, Axillar surgery are 0.00019, 2.08E-05, 1.63E-09, 2.27E-12, 2.20E-16, 3.89E-07, 6.36E-06, 8.67E-05, 2.33E-05, 5.10E-07, 0.00012, 8.29E-05, 4.13E-06, 0.0003, 2.80E-15, 2.00E-07, 5.44E-10. Abbreviations: AND, axillary lymph node dissection; BMI, body mass index; DCIS, ductal carcinoma in situ; ER, estrogen receptor; LVI, lymphovascular invasion; NET, neoadjuvant endocrine therapy; NRT, neoadjuvant radiotherapy; NST, no special type; PR, progesterone receptor; SND, sentinel lymph node dissection; TNBC: triple negative breast cancer. “n” denotes the number of patients. Categorical variables are expressed as absolute numbers (percentages in brackets). Continuous variables are expressed as mean values, with the standard deviation in brackets.

Supplementary Table 2: Patient's and tumor characteristics of the cohort of 313 patients with invasive sBBCs.

	level	n
**Patient level**		313
**Age**		60.8 (12.8)
**Age class**	<40	13 (4.2)
	[40-50[	56 (17.9)
	[50-60[	81 (26.0)
	[60-70[	86 (27.6)
	>=70	76 (24.4)
**BMI**		25.9 (5.1)
**BMI class**	<18.5	7 (2.4)
	18.5-24.9	147 (49.8)
	25-29.9	83 (28.1)
	>=30	58 (19.7)
**Previous pregnancy**	No	50 (16.8)
	Yes	248 (83.2)
**Menopausal status**	Premenopausal	89 (29.7)
	Postmenopausal	211 (70.3)
**Familial history breast / ov. cancer**	No	101 (42.3)
	Yes	138 (57.7)
**Research hereditary predisposition**	No	40 (44.4)
	Yes	50 (55.6)
**BRCA mutation**	No	37 (74.0)
	Yes	13 (26.0)
**First treatment**	Chemotherapy	52 (16.6)
	NET / NRT / other	21 (6.7)
	Surgery	240 (76.7)
**Chemotherapy**	No	146 (46.6)
	Yes	167 (53.4)
Chemotherapy regimen	*Anthra-taxanes*	*138 (44.1)*
	Anthracyclines	*22 (7.0)*
	Taxanes	*7 (2.2)*
**Endocrine therapy**	No	39 (12.5)
	Yes	274 (87.5)
**Targeted therapy**	No	286 (91.4)
	Yes	27 (8.6)
**Radiotherapy**	No	52 (16.6)
	Yes	261 (83.4)
**Tumor level**		626
**Diagnostic modality**	Non-palpable	355 (59.1)
	Palpable	246 (40.9)
**Clinical size**		31.4 (21.6)
**Clinical T stage**	T0 or pTis	248 (39.9)
	T1	173 (27.9)
	T2	147 (23.7)
	T3	31 (5.0)
	T4	22 (3.5)
**Clinical N stage**	cN0	552 (88.5)
	cN1/N2/N3	72 (11.5)

**Pathological T stage**	pT1	357 (73.8)
	pT2	111 (22.9)
	pT3	15 (3.1)
	pT4	1 (0.2)
**BC subtype**	luminal	538 (87.6)
	TNBC	44 (7.2)
	HER2+	32 (5.2)
**ER status**	negative	56 (9.1)
	positive	562 (90.9)
**PR status**	negative	145 (24.3)
	positive	452 (75.7)
***HER2* status**	negative	582 (94.8)
	positive	32 (5.2)
**ER positivity (intensity)**	+	41 (7.5)
	++	156 (28.4)
	+++	352 (64.1)
**ER positivity (%)**		78.2 (30.6)
**PR positivity (intensity)**	+	58 (12.2)
	++	149 (31.3)
	+++	269 (56.5)
**PR positivity (%)**		51.2 (38.6)
**DCIS component**	DCIS	414 (68.2)
	No DCIS	193 (31.8)
**Histological size**		17.6 (13.0)
**pN status**	0	329 (67.6)
	[1-3]	111 (22.8)
	4 and more	47 (9.7)
**Grade**	1	181 (29.8)
	2	311 (51.2)
	3	116 (19.1)
**Lymphovascular invasion**	No	376 (80.0)
	Yes	94 (20.0)
**Histological type**	lobular	114 (18.4)
	NST	443 (71.3)
	others	64 (10.3)
**Multifocality**	No / NA	438 (70.0)
	Yes	188 (30.0)
**Breast surgery**	Lumpectomy	359 (57.3)
	Mastectomy	259 (41.4)
	No surgery	8 (1.3)
**Axillar surgery**	AND	359 (57.9)
	SND	248 (40.0)
	No axillary sampling	13 (2.1)

Missing data: Age, n=1; Age class, n=1; BMI, n=18; BMI class, n=18; Previous pregnancy, n=15; Menopausal status, n=13; Familial history breast / ov. cancer, n=74; Research hereditary predisposition, n=223; BRCA mutation, n=263
Diagnostic modality, n=25; clinical size, n=288; clinical T stage, n=5; clinical N stage, n=2; pathological T stage, n=142; breast cancer subtype, n=12; ER status, n=8; PR status, n=29; HER2 status, n=12; intensity of ER positivity, n=77; percentage of ER positivity, n=16; intensity of PR positivity, n=150; percentage of PR positivity, n=37; DCIS component, n=19; pN status, n=139; grade, n=45; lymphovascular invasion, n=156; histological type, n=5; axillar surgery, n=6.
Abbreviations: DCIS, ductal carcinoma in situ; ER, estrogen receptor; NET, neoadjuvant endocrine therapy; NRT, neoadjuvant radiotherapy; NST, no special type; PR, progesterone receptor; SND, sentinel lymph node dissection; TNBC: triple negative breast cancer. "n" denotes the number of patients. Categorical variables are expressed as absolute numbers (percentages in brackets). Continuous variables are expressed as mean values, with the standard deviation in brackets.

Supplementary Table 3 : Patient's and tumor characteristics according to the presence of a germline genetic predisposition

		Overall	Germline genetic predisposition	No genetic predisposition	*p*
Patient level		313	13 (4.2)	37 (11.8)	
**Age**		60.8 (12.8)	43.6 (8.2)	50.8 (8.8)	**0.012**
**BMI class**	18.5-24.9	147 (49.8)	5 (41.7)	19 (52.8)	NaN
	<18.5	7 (2.4)	0 (0.0)	0 (0.0)	
	25-29.9	83 (28.1)	6 (50.0)	13 (36.1)	
	>=30	58 (19.7)	1 (8.3)	4 (11.1)	
**Previous pregnancy**	No	50 (16.8)	4 (30.8)	6 (16.2)	0.468
	Yes	248 (83.2)	9 (69.2)	31 (83.8)	
**Menopausal status**	Premenopausal	89 (29.7)	11 (84.6)	23 (63.9)	0.299
	Postmenopausal	211 (70.3)	2 (15.4)	13 (36.1)	
**Tumor level**		626	26 (4.2)	74 (11.8)	
		626	26 (4.2)	74 (11.8)	
**Diagnostic modality**	Non-palpable	355 (59.1)	8 (33.3)	45 (62.5)	**0.024**
	Palpable	246 (40.9)	16 (66.7)	27 (37.5)	
**Clinical T stage**	T0 or pTis	248 (39.9)	8 (30.8)	35 (47.9)	**0.033**
	T1	173 (27.9)	4 (15.4)	22 (30.1)	
	T2	147 (23.7)	12 (46.2)	12 (16.4)	
	T3	31 (5.0)	1 (3.8)	1 (1.4)	
	T4	22 (3.5)	1 (3.8)	3 (4.1)	
**Clinical N stage**	cN0	552 (88.5)	19 (73.1)	64 (86.5)	0.207
	cN1/N2/N3	72 (11.5)	7 (26.9)	10 (13.5)	
**BC subtype**	luminal	538 (87.6)	11 (44.0)	61 (83.6)	**<0.001**
	TNBC	44 (7.2)	14 (56.0)	5 (6.8)	
	HER2+	32 (5.2)	0 (0.0)	7 (9.6)	
**Concordance**	Concordant	512 (84.8)	22 (91.7)	52 (72.2)	0.092
	Discordant	92 (15.2)	2 (8.3)	20 (27.8)	
**DCIS component**	DCIS	414 (68.2)	15 (60.0)	53 (73.6)	0.304
	No DCIS	193 (31.8)	10 (40.0)	19 (26.4)	
**Tumor cellularity (%)**		65.8 (17.2)	72.9 (12.0)	64.2 (19.3)	0.137
**Tumor grade**	1	181 (29.8)	1 (4.2)	19 (25.7)	**<0.001**
	2	311 (51.2)	7 (29.2)	38 (51.4)	
	3	116 (19.1)	16 (66.7)	17 (23.0)	
**Intratumoral TILs (%)**		6.5 (7.6)	11.9 (11.1)	8.0 (7.4)	0.19
**Str TILs (%)**		13.3 (12.5)	16.9 (14.1)	15.5 (12.5)	0.75
**Histological type**	lobular	114 (18.4)	1 (3.8)	10 (13.5)	0.365
	NST	443 (71.3)	23 (88.5)	57 (77.0)	
	others	64 (10.3)	2 (7.7)	7 (9.5)	
**Multifocality**	No / NA	438 (70.0)	18 (69.2)	46 (62.2)	0.683
	Yes	188 (30.0)	8 (30.8)	28 (37.8)	

Statistical tests were chi-square tests or fisher tests when indicated for categorical variables and Wilcoxon-tests or Kruskall-Wallis for quantitative variables. All tests were two sided. Exact p-values for the variables breast cancer subtype and tumor grade are 3.795e-07 and 0.0003 respectively. “n” denotes the number of patients. Categorical variables are expressed as absolute numbers (percentages in brackets). Continuous variables are expressed as mean values, with the standard deviation in brackets.

Supplementary Table 4 : Concordance between left and right tumor's characteristics.

	concordance	Kappa	*p*	Kendall	*p*
Diagnostic modality	62.5	0.23	**<0.001**		
Clinical size				0.73	**<0.001**
Clinical T stage	39.5	0.14	**<0.001**		
Clinical N stage	83	0.16	0.08		
Pathological T stage	62.1	0.06	0.21		
BC subtype	84.8	0.32	**<0.001**		
ER status	91.1	0.46	**<0.001**		
PR status	74.7	0.31	**<0.001**		
HER2 status	90.7	0.08	0.33		
ER positivity (intensity)	65.2	0.29	**<0.001**		
ER positivity (%)				0.71	**<0.001**
PR positivity (intensity)	55.7	0.21	**<0.001**		
PR positivity (%)				0.68	**<0.001**
Intratumoral TILs (%)				0.69	**<0.001**
Str TILs (%)				0.67	**<0.001**
DCIS component	69.7	0.3	**<0.001**		
pN status	52.7	0.02	0.4		
Grade	48.3	0.15	**<0.001**		
Lymphovascular invasion	73.7	0.17	**0.04**		
Histological type	62.8	0.17	**<0.001**		
Multifocality	62.3	0.1	0.06		
Breast surgery	68.4	0.37	**<0.001**		
Axillar surgery	54.8	0.1	**0.03**		

Abbreviations: BMI, body mass index; DCIS, ductal carcinoma in situ; ER, estrogen receptor; intratumoral TIL, intratumoral tumor infiltrating lymphocytes; PR, progesterone receptor; str TIL, stromal tumor infiltrating lymphocytes; TNBC: triple negative breast cancer. In case of categorical variables, the kappa coefficient was computed as a measure of concordance between the left and the right side; otherwise in case of numeric or integer variables, the kendall test was used. The exact p-values for the test for the variables Clinical T stage, BC subtype, ER status, PR status, ER positivity (intensity), PR positivity (intensity), DCIS component, Grade, Histological type, Breast surgery are p=7.6e-05, p =7.07e-05, p =0.001, p =0.0002, p =2.4e-05, p=3.5e-06, p =0.0003, P =2.7e-06, p =0.0006, p=0.0004, p =2.2e-11 respectively. The exact p-values for the variables Clinical size, ER positivity (%), PR positivity (%), Intratumoral TILs (%), Str TILs (%) are p=1.4e-06, p=1,2e-14, p=3,9e-10, p=1,4e-05, p=0,0001 respectively.

Supplementary Table 5: Association between patient's tumor's characteristics and immune infiltration (stromal TIL levels), univariate and multivariate analysis.

		Str TILs				Univariate		Multivariate	
Variable	levels	n	mean	median	*p*	exp(Beta) [95% CI]	*p-value*	exp(Beta) [95% CI]	*p-value*
**Age class**	<40	9	23.9	25	**0.017**	1	0.14		
	[40-50[	46	13	10		-10.85 [-19.77--1.93]			
	[50-60[	76	13.5	10		-10.38 [-19--1.75]			
	[60-70[	80	12.5	10		-11.35 [-19.96--2.75]			
	>=70	64	12.6	10		-11.28 [-19.99--2.57]			
**BMI class**	18.5-24.9	137	14.6	10	0.776	1	0.41		
	<18.5	6	12.5	10		-2.08 [-12.37-8.22]			
	25-29.9	80	12.8	10		-1.78 [-5.25-1.7]			
	>=30	50	11.2	10		-3.36 [-7.44-0.72]			
**Menopausal status**	Premenopausal	77	14.5	10	0.137	1	0.2		
	Postmenopausal	187	12.3	10		-2.16 [-5.43-1.11]			
**BRCA mutation**	No	26	15.5	10	0.823	1	0.75		
	Yes	16	16.9	10		1.34 [-6.83-9.51]			
**Diagnostic modality**	Non-palpable	166	11.2	10	**0.006**	1	**0.00072**	1	**0.04**
	Palpable	111	16.4	10		5.1 [2.15-8.06]		3.04 [0.14 - 5.95]	
**Clinical T stage**	T0 or pTis	105	11.4	10	0.259	1	0.11		
	T1	72	13.4	10		2.01 [-1.74-5.77]			
	T2	67	16.3	10		4.86 [1.03-8.7]			
	T3	16	15.8	10		4.39 [-2.19-10.98]			
	T4	13	10.4	5		-1.03 [-8.25-6.18]			
**Clinical N stage**	cN0	242	12.5	10	0.114	1	**0.0019**		
	cN1/N2/N3	34	19.5	15		7.05 [2.61-11.48]			
**Pathological T stage**	pT1	149	11	10	0.081	1	**0.0026**		
	pT2	49	18	10		7.06 [3.27-10.85]			
	pT3	4	8.7	7.5		-2.23 [-13.88-9.42]			
	pT4	1	5	5		-5.98 [-29.05-17.09]			
**DCIS component**	DCIS	180	12.4	10	0.687	1	0.14		
	No DCIS	96	14.8	10		2.35 [-0.75-5.45]			
**Tumor cellularity (%)**						0.1 [0.01-0.19]	**0.022**		
**pN status**	0	146	11.1	10	**<0.001**	1	**<0.0001**		
	[1-3]	41	13.1	10		2.02 [-1.95-5.99]			
	4 and more	16	25.6	22.5		14.57 [8.65-20.49]			
**Grade**	1	78	9.1	10	**<0.001**	1	**<0.0001**	1	**<0.001**
	2	138	11.9	10		2.82 [-0.41-6.05]		2.08 [-1.17- 5.33]	
	3	58	22.4	17.5		13.38 [9.43-17.33]		10.6 [6.36 - 14.8]	
**Lymphovascular invasion**	No	156	11	10	**<0.001**	1	**0.00075**		
	Yes	40	17.8	15		6.71 [2.81-10.61]			
**Histological type**	lobular	49	10.3	5	**0.021**	1	0.13		
	NST	205	14.2	10		3.82 [-0.07-7.72]			
	others	23	11.7	5		1.39 [-4.8-7.58]			
**Multifocality**	No / NA	190	13.5	10	0.71	1	0.62		
	Yes	87	12.7	10		-0.81 [-4-2.37]			
**BC subtype**	luminal	240	11.8	10	**<0.001**	1	**<0.0001**		
	TNBC	17	22.9	20		11.1 [5.21-16.98]			
	HER2+	16	23.9	17.5		12.03 [5.97-18.09]			
**Concordance BC subtype**	Concordant	226	11.8	10	**<0.001**	1	**<0.0001**	1	**0.002**
	Discordant	45	20.9	15		9.06 [5.19-12.93]		6.07 [2.23 - 9.90]	
Interaction concordance						1	0.6		
/subtype	Discordant * TNBC					-1.71 [-14.7, 11.3]			
	Discordant * HER2+					-7.76 [-22.3, 6.78]			

Abbreviations: BMI, body mass index; DCIS, ductal carcinoma in situ; ER, estrogen receptor; NST, no special type; PR, progesterone receptor; TNBC: triple negative breast cancer. Statistical tests were Wilcoxon-tests/t-tests or anova/Kruskall Wallis tests when indicated, all tests were two sided. The exact p-values for the test for the variables pN status, grade, LVI, BC subtype, concordance, p= 1.58E-05, p= 2.33E-10, p= 0.0009, p= 2.51E-06, p=0.0003). In univariate analysis, exact p-values for nodal status, grade, BC subtype, concordance are: p: 2.76e-06, p= 1.79e-10, p= 2.506e-06, p= 6.71e-06 respectively. In multivariate analysis, exact p-values are grade: p= 1.29e-06 respectively.

Supplementary Table 6: Association between patient's tumor's characteristics and immune infiltration (intratumoral TIL levels), univariate and multivariate analysis.

		Intratumoral TILs				Univariate		Multivariate	
Variable	levels	n	mean	median	*p*	OR	*p-value*	exp(Beta) [95% CI]	*p-value*
**Age class**	<40	9	16.3	15	**0.011**	1	**0.0016**		
	[40-50[	44	6.2	3		-10.17 [-15.51--4.84]			
	[50-60[	76	6.6	5		-9.69 [-14.83--4.54]			
	[60-70[	80	5.4	5		-10.88 [-16.01--5.75]			
	>=70	64	6.5	4		-9.85 [-15.04--4.65]			
**BMI class**	18.5-24.9	137	7.1	5	0.688	1	0.35		
	<18.5	6	5.7	5		-1.42 [-7.67-4.83]			
	25-29.9	78	6.7	5		-0.42 [-2.55-1.7]			
	>=30	50	4.8	5		-2.25 [-4.72-0.23]			
**Menopausal status**	Premenopausal	75	7.3	5	0.204	1	0.18		
	Postmenopausal	187	5.9	5		-1.38 [-3.4-0.65]			
**BRCA mutation**	No	26	8	5	0.557	1	0.18		
	Yes	14	11.9	12.5		3.93 [-1.84-9.69]			
**Diagnostic modality**	Non-palpable	165	5.1	3	**0.003**	1	**0.00024**		
	Palpable	110	8.5	5		3.36 [1.57-5.16]			
**Clinical T stage**	T0 or pTis	105	5.3	5	0.333	1	**0.039**		
	T1	72	6.2	5		0.92 [-1.35-3.19]			
	T2	65	8.8	5		3.57 [1.23-5.91]			
	T3	16	7.7	5		2.41 [-1.57-6.39]			
	T4	13	4.9	3		-0.35 [-4.71-4.01]			
**Clinical N stage**	cN0	241	6.1	5	0.34	1	**0.023**		
	cN1/N2/N3	33	9.3	5		3.2 [0.45-5.94]			
**Pathological T stage**	pT1	149	5.3	5	0.082	1	**0.031**		
	pT2	49	8.8	5		3.47 [1.14-5.81]			
	pT3	4	4.7	3.5		-0.55 [-7.73-6.62]			
	pT4	1	3	3		-2.3 [-16.51-11.9]			
**DCIS component**	DCIS	180	5.9	5	0.913	1	0.1		
	No DCIS	94	7.5	5		1.57 [-0.33-3.46]			
**Tumor cellularity (%)**						0.07 [0.02-0.12]	**0.011**		
**pN status**	0	146	5.4	3	**<0.001**	1	**0.00035**		
	[1-3]	41	6.2	5		0.8 [-1.65-3.26]			
	4 and more	16	12.8	10		7.44 [3.78-11.1]			
**Grade**	1	78	4.2	3	**<0.001**	1	**<0.0001**	1	**<0.001**
	2	138	5.6	5		1.47 [-0.51-3.44]		1.02 [-0.89 - 2.94]	
	3	56	11.9	10		7.76 [5.31-10.2]		6.88 [4.29 - 9.46]	
**Lymphovascular invasion**	No	156	5.4	3	**<0.001**	1	**0.017**		
	Yes	40	8.4	5		2.95 [0.54-5.37]			
**Histological type**	lobular	49	5.2	2	**0.02**	1	**0.39**		
	NST	203	6.8	5		1.65 [-0.72-4.03]			
	others	23	6.2	3		1.05 [-2.72-4.82]			
**Multifocality**	No / NA	189	6.8	5	0.723	1	0.28		
	Yes	86	5.7	5		-1.06 [-3-0.88]			
**BC subtype**	luminal	240	5.7	5	**<0.001**	1	**<0.0001**	1	
	TNBC	15	12.7	10		7.07 [3.29-10.85]		13.3 [6.44 - 20.1]	**<0.001**
	HER2+	16	11.8	10		6.15 [2.48-9.82]		5.47 [-1.27 - 12.2]	
**Concordance BC subtype**	Concordant	224	5.8	5	**0.007**	1	**0.00095**	1	**0.028**
	Discordant	45	9.8	5		4 [1.63-6.37]		3.41 [0.37- 6.46]	
Interaction concordance						1	**<0.001**	1	**<0.001**
/subtype	Discordant * TNBC					-16.9 [-25.7, -8.17]		-18.2 [-26.5- -9.85]	
	Discordant * HER2+					-3.51 [-12.2, 5.15]		-4.22 [-12.5- 4.04]	

Abbreviations: BMI, body mass index; DCIS, ductal carcinoma in situ; ER, estrogen receptor; NST, no special type; PR, progesterone receptor; TNBC: triple negative breast cancer. Statistical tests were Wilcoxon-tests/t-tests or anova/Kruskall Wallis tests when indicated, all tests were two sided. The exact p-values for the test for the variables pN status, grade, LVI, BC subtype, p= 0.0005, p= 2.22E-09, p= 0.0004, p= 1.71E-05). In univariate analysis, exact p-values for grade, BC subtype, interaction concordance*subtype are p= 2.217e-09, p= 1.709e-05, p= 0.0007. In multivariate analysis, exact p-values for grade, BC subtype, interaction concordance*subtype are: p= 3.31e-07, p= 0.0002, p=2.5e-05 respectively.

Supplemental Table 7: Association between patient's tumor's characteristics and pathological complete response (pCR), univariate and multivariate analysis.

					Univariate			Multivariate		
Variable name	Level	n=	pCR	% pCR	OR	OR(95%)	p	OR	OR(95%)	p
**Age class**	<40	12	6	50%	1					
	[40-50[	32	5	15.60%	0.19	[0.04 - 0.8]	**0.026**			
	[50-60[	34	6	17.60%	0.21	[0.05 - 0.89]	**0.035**			
	[60-70[	22	3	13.60%	0.16	[0.03 - 0.78]	**0.03**			
	>=70	30	1	3.30%	0.03	[0 - 0.25]	**0.004**			
**BMI class**	18.5-24.9	61	9	15%						
	<18.5	0	0	NaN %						
	25-29.9	41	10	24%						
	>=30	30	3	10.00%						
**Previous pregnancy**	No	12	3	25.00%	1					
	Yes	117	19	16.20%	0.58	[0.16 - 2.8]	0.447			
**Menopausal status**	Premenopausal	52	12	23.10%	1					
	Postmenopausal	78	10	12.80%	0.49	[0.19 - 1.24]	0.131			
***BRCA* mutation**	No	20	4	20%	1					
	Yes	12	7	58.30%	5.6	[1.21 - 30.31]	**0.033**			
**Diagnostic modality**	Non-palpable	55	10	18.20%	1					
	Palpable	73	12	16.40%	0.89	[0.35 - 2.27]	0.796			
**Clinical T stage**	T0 or pTis	34	7	21%	1					
	T1	14	3	21%	1.05	[0.2 - 4.59]	0.948			
	T2	48	7	14.60%	0.66	[0.2 - 2.13]	0.478			
	T3	17	3	18%	0.83	[0.16 - 3.5]	0.803			
	T4	19	2	10.50%	0.45	[0.06 - 2.15]	0.358			
**Clinical N stage**	cN0	100	17	17.00%	1					
	cN1/N2/N3	31	5	16.10%	0.94	[0.29 - 2.64]	0.91			
**DCIS component**	DCIS	35	6	17.10%	1					
	No DCIS	90	16	17.80%	1.05	[0.39 - 3.15]	0.933			
**Tumor cellularity**		72	11	7.90%			0.077			
**Tumor grade**	1	18	1	5.60%	1					
	2	61	7	11.50%	2.2	[0.36 - 42.72]	0.474			
	3	43	13	30.20%	7.37	[1.29 - 139.66]	0.065			
**Intratumoral TILs (%)**		72	11	8%	1.08	[1.01 - 1.16]	**0.026**			
**str TILs (%)**		74	12	9%	1.06	[1.01 - 1.1]	**0.012**	1.05	[1 - 1.1]	**0.038**
**Histological type**	lobular	17	0	0%						
	NST	102	21	20.60%						
	others	8	1	13%						
**Multifocality**	No / NA	103	14	13.60%	1					
	Yes	29	8	28%	2.42	[0.87 - 6.45]	0.08			
**BC subtype**	luminal	92	8	8.70%	1			1		
	TNBC	27	11	40.70%	7.22	[2.55 - 21.51]	**<0.001**	10.2	[1.32 - 88.86]	**0.026**
	HER2+	9	3	33.30%	5.25	[0.97 - 24.43]	**0.038**	2.29	[0.19 - 27.6]	0.498
**Concordance**	Concordant	86	12	14%	1			1		
	Discordant	38	10	26.30%	2.2	[0.84 - 5.69]	0.102	3.41	[0.46 - 23.87]	0.208
	Interaction subtype / concordance						***0.02***			***0.03***

Abbreviations: BMI, body mass index; DCIS, ductal carcinoma in situ; ER, estrogen receptor; NST, no special type; PR, progesterone receptor; TNBC: triple negative breast cancer. Statistical tests were chi-square or fisher tests when indicated, all tests were two sided. In univariate analysis, exact p-values for BC subtype is p=0.0002.

Supplementary Table 8: Association between patient's tumor's characteristics and relapse-free survival (RFS), univariate and multivariate analysis.

				Univariable			Multivariable		
Variable name	Level	n=	ev	HR	95%CI	*p*	HR	95%CI	*p*
**Age class**	<40	13	4	1		0.21			
	[40-50[	56	9	0.54	[0.17 - 1.77]				
	[50-60[	81	16	0.62	[0.21 - 1.85]				
	[60-70[	86	12	0.45	[0.14 - 1.39]				
	>=70	76	17	0.99	[0.33 - 2.97]				
**BMI class**	18.5-24.9	147	27	1		0.44			
	<18.5	7	0	0	[0 - Inf]				
	25-29.9	83	18	1.45	[0.79 - 2.64]				
	>=30	58	10	1.11	[0.54 - 2.3]				
**Previous pregnancy**	No	50	13	1		0.12			
	Yes	248	42	0.61	[0.33 - 1.14]				
**Menopausal status**	Premenopausal	89	18	1		0.82			
	Postmenopausal	211	38	0.94	[0.53 - 1.64]				
***BRCA* mutation**	No	37	8	1		0.49			
	Yes	13	4	1.52	[0.46 - 5.06]				
**Diagnostic modality**	Non-palpable	355	59	1		0.04			
	Palpable	246	54	1.46	[1.01 - 2.11]				
**Clinical T stage**	T0 or pTis	248	39	1		**<0.01**	1.71	[0.91 - 3.2]	0.094
	T1	173	23	0.84	[0.5 - 1.41]		0.98	[0.58 - 1.68]	0.95
	T2	147	33	1.58	[0.99 - 2.51]		1.42	[0.85 - 2.38]	0.185
	T3	31	8	2.1	[0.98 - 4.5]		1.64	[0.74 - 3.64]	0.227
	T4	22	13	4.88	[2.61 - 9.16]		5.79	[2.99 - 11.22]	**<0.001**
**Clinical N stage**	cN0	552	92	1		**<0.01**			
	cN1/N2/N3	72	24	2.54	[1.62 - 3.98]				
**DCIS component**	DCIS	414	71	1		0.19			
	No DCIS	193	38	1.3	[0.88 - 1.93]				
**Tumor grade**	1	181	22	1		**<0.01**	1	-	-
	2	311	50	1.54	[0.93 - 2.55]		1.73	[1.03 - 2.91]	**0.039**
	3	116	37	2.9	[1.71 - 4.92]		2.22	[1.19 - 4.16]	**0.012**
**Intratumoral TILs (%)**		275	39	1	[0.96 - 1.04]	0.89			
**Str TILs (%)**		277	41	0.99	[0.97 - 1.02]	0.67			
**Histological type**	lobular	114	21	1		0.79			
	NST	443	80	0.94	[0.58 - 1.52]				
	others	64	14	1.14	[0.58 - 2.24]				
**Multifocality**	No / NA	438	75	1		0.16			
	Yes	188	41	1.31	[0.9 - 1.92]				
**BC subtype**	luminal	538	93	1		**<0.01**	1	-	**-**
	TNBC	44	17	2.64	[1.57 - 4.43]		1.71	[0.91 - 3.2]	**0.094**
	HER2+	32	4	0.64	[0.24 - 1.75]		0.34	[0.1 - 1.1]	**0.071**
**Concordance**	Concordant	512	96	1		0.58			
	Discordant	92	16	0.86	[0.51 - 1.46]				
Interaction subtype / concordance						0.46			

Abbreviations: BMI, body mass index; DCIS, ductal carcinoma in situ; ER, estrogen receptor; NST, no special type; PR, progesterone receptor; TNBC: triple negative breast cancer. Statistical tests were Wald test for the p-value for univariate and multivariate cox proportional hazard models, Exact p-values for Clinical T stage, Clinical N stage, Tumor grade and BC subtype are p= 9e-06, p= 4e-05, p= 5e-05, p= 8e-04 respectively.

Supplementary Table 9: Patient's and tumor's characteristics of the 6 patients and 20 samples with NGS data.

patient number	Patient1		Patient2		Patient3		Patient4		Patient5		Patient6			
Side	Right	Left	Right	Left	Right	Left	Right	Left	Right	Left	Right	Right	Left	Left
Clinical T stage	T2	T2	T2	T2	T2	T1	T2	T4d	T2	T1	T2	T2	T3	T3
Clinical N stage	N0	N3	N0	N0	N0	N0	N0	N1	N0	N0	N0	N0	N3	N3
Age	42.5 y.o.		38.1 y.o.		51 y.o.		72.4 y.o.		69.5 y.o.		46 y.o.			
Menopausal status	Premenopausal		Premenopausal		Premenopausal		Postmenopausal		Postmenopausal		Premenopausal			
BRCA germline mutation	**BRCA1mut**		**BRCA1mut**		**BRCAwt**		**BRCA2mut**		**BRCAwt**		**BRCA2mut**			
Mutation type	c.4484G>T/p.Arg1495Met		c.1380dupA/p.Phe461IlefsX19				c.7406dup, p.(Thr2471Hisfs*4)				c.1310_1313del p.(Lys437Ilefs*22)			
Pre-NAC parameters														
Subtype	TNBC	TNBC	TNBC	TNBC	luminal B	luminal A	luminal B	luminal B	TNBC	luminal B	luminal A	luminal B	luminal B	luminal B
ER status	ER neg	ER neg	ER neg	ER neg	ER ++ 90%	ER ++ 90%	ER +++ 95%	ER ++ 90%	ER NA 5%	ER + 25%	ER ++ 80%	ER + 70%	ER + 80%	ER + 80%
PR status	PR neg	PR neg	PR neg	PR neg	PR + 60%	PR + 90%	PR ++ 80%	PR neg	PR neg	PR neg	PR ++ 60%	PR + 15%	PR + 15%	PR + 5%
HER2 status	HER2 neg	HER2 neg	HER2 neg	HER2 neg	HER2 neg	HER2 neg	HER2 neg	HER2 neg	HER2 neg	HER2 neg	HER2 neg	HER2 neg	HER2 neg	HER2 neg
Grade	3	3	3	3	2	1	2	3	3	3	2	3	3	3
Mitotic index	100	100	25	45	7	0	14	100	29	35	5	14	30	42
KI67	NA	NA	NA	NA	40	15	25	25	30	80	10	10	30	30
Histological type	NST	NST	NST	NST	NST	lobular	NST	NST	NST	NST	NST	NST	NST	NST
Str TILs (%)	5	5	30	25	5	20	50	15	20	60	5	25	25	20
Intratumoral TILs (%)	NA	NA	40	20	2	10	30	10	5	20	1	2	5	2
Chemotherapy regimen	Anthra-taxanes		Anthra-taxanes		Anthra-taxanes		Anthra-taxanes		Anthra-taxanes		Anthra-taxanes			
Breast surgery	Mastectomy	Mastectomy	Mastectomy	Mastectomy	Lumpectomy	Lumpectomy	Lumpectomy	Mastectomy	Lumpectomy	Lumpectomy	Mastectomy	Mastectomy	Mastectomy	Mastectomy
Axillar surgery	AND	AND	AND	SND	SND	SND	AND	AND	SND	AND	AND	AND	AND	AND
Post-NAC parameters														
RCB (class)	pCR	RCB-II	pCR	pCR	RCB-II	RCB-II	RCB-II	pCR	RCB-II	pCR	RCB-II	RCB-II	pCR	pCR
Number of positive nodes (post NAC)	0	1	0	0	0	0	2	0	0	0	1	1	0	0
Lymphovascular invasion (post NAC)	no	no	no	no	yes	no	yes	NA	no	no	no	no	no	no
Mitotic index (post NAC)	NA	26	NA	NA	1	0	12	NA	11	NA	NA	1	NA	NA
Str TILs (%)	10	20	1	1	10	5	25	15	25	10	5	5	5	5
Intratumoral TILs (%)	NA	10	NA	NA	2	2	10	NA	5	NA	NA	1	NA	NA
Radiotherapy	Yes	Yes	Yes	Yes	Yes	Yes	Yes	Yes	Yes	Yes	Yes	Yes	Yes	Yes
Endocrine therapy	No		No		Yes		Yes		No		Yes			
Survival outcomes														
Local relapse	No	Yes	No	No	No	No	No	No	No	No	No	No	No	No
comment		*Chest wall*												
Distant metastasis	Yes		No		No		No		No		No			
Deaths	Yes		No		No		No		No		No			
Delay diagnosis to RFS event (months)	18		131		90		95		76		78			

Abbreviations: AND, axillary lymph node dissection; ER, estrogen receptor; intratumoral TIL, intratumoral tumor infiltrating lymphocytes; L, left; NAC, neoadjuvant chemotherapy; PR, progesterone receptor; R, right; RCB, residual cancer burden; SND, sentinel lymph node dissection; str TIL, stromal tumor infiltrating lymphocytes; wt, wild type.
