# Supplementary material for: Evolution of synchronous female bilateral breast cancers and response to treatment
Source: Nat Med. 2023 Mar 6;29(3):646–55. doi: 10.1038/s41591-023-02216-8 (PMC10033420; doi:10.1038/s41591-023-02216-8)
Supplement: Supplementary file 2 — Reporting Summary [file 41591_2023_2216_MOESM2_ESM.pdf]

nature portfolio

Corresponding author(s): Fabien Reyal & Joshua Waterfall

Last updated by author(s): Nov 24, 2022

Reporting Summary

Nature Portfolio wishes to improve the reproducibility of the work that we publish. This form provides structure for consistency and transparency in reporting. For further information on Nature Portfolio policies, see our Editorial Policies and the Editorial Policy Checklist.

Statistics

For all statistical analyses, confirm that the following items are present in the figure legend, table legend, main text, or Methods section.

n/a	Confirmed	
☐	☒	The exact sample size (*n*) for each experimental group/condition, given as a discrete number and unit of measurement
☐	☒	A statement on whether measurements were taken from distinct samples or whether the same sample was measured repeatedly
☐	☒	The statistical test(s) used AND whether they are one- or two-sided<br>*Only common tests should be described solely by name; describe more complex techniques in the Methods section.*
☐	☒	A description of all covariates tested
☐	☒	A description of any assumptions or corrections, such as tests of normality and adjustment for multiple comparisons
☐	☒	A full description of the statistical parameters including central tendency (e.g. means) or other basic estimates (e.g. regression coefficient) AND variation (e.g. standard deviation) or associated estimates of uncertainty (e.g. confidence intervals)
☐	☒	For null hypothesis testing, the test statistic (e.g. *F*, *t*, *r*) with confidence intervals, effect sizes, degrees of freedom and *P* value noted<br>*Give P values as exact values whenever suitable.*
☒	☐	For Bayesian analysis, information on the choice of priors and Markov chain Monte Carlo settings
☒	☐	For hierarchical and complex designs, identification of the appropriate level for tests and full reporting of outcomes
☐	☒	Estimates of effect sizes (e.g. Cohen's *d*, Pearson's *r*), indicating how they were calculated

Our web collection on statistics for biologists contains articles on many of the points above.

Software and code

Policy information about availability of computer code

Data collection	REDCap* 12.4.14, MACRO version 3
Data analysis	R software version 4.2.1,SuperFreq v. 1.3.2,Burrows-Wheeler Aligner v0.7.5a, package scikit-learn version 0.21.3,VEP (version 104), package musicatk (version 1.0.0),TopHat v.2.0.621, Picard v.1.97, DESeq2(1.22.1). Codes are available at https://github.com/rt2lab/bc_bilat_neo

For manuscripts utilizing custom algorithms or software that are central to the research but not yet described in published literature, software must be made available to editors and reviewers. We strongly encourage code deposition in a community repository (e.g. GitHub). See the Nature Portfolio guidelines for submitting code & software for further information.

Data

Policy information about availability of data

All manuscripts must include a data availability statement. This statement should provide the following information, where applicable:

- Accession codes, unique identifiers, or web links for publicly available datasets
- A description of any restrictions on data availability
- For clinical datasets or third party data, please ensure that the statement adheres to our policy

The genomic and transcriptomic data generated during the study are available as preprocessed files at the following link https://github.com/rt2lab/bc_bilat_neo (folder data/external).Raw sequence data have been deposited at the European Genome-phenome Archive (EGA), which is hosted by the EBI and the CRG, under accession number EGAS00001006910.

Human research participants

Policy information about studies involving human research participants and Sex and Gender in Research.

Reporting on sex and gender	Sex/gender were considered in the study design and only female patients were included in the study.
Population characteristics	We identified a cohort of 17575 patients with non metastatic breast cancer treated at the institut Curie (Paris and Saint Cloud, France) between 2005 and 2012 in the institutional database (median age 58.2 y.o.).
Recruitment	All patients undergoing routine care in Institut Curie are asked to fill an inform consent to specify whether they accept to participate in further medical or scientific research including research on clinical and pathological data, and research on tumor samples including genomic analyses. Biases in the study include differences between patients refusing data and samples reuse for research purposes and patients consenting to it.
Ethics oversight	This study was approved by the Breast Cancer Study Group and by the Institutional Review Board of Institut Curie and was conducted in accordance with institutional and ethical rules regarding research on tissue specimens and patients.

Note that full information on the approval of the study protocol must also be provided in the manuscript.

Field-specific reporting

Please select the one below that is the best fit for your research. If you are not sure, read the appropriate sections before making your selection.

☒ Life sciences ☐ Behavioural & social sciences ☐ Ecological, evolutionary & environmental sciences

For a reference copy of the document with all sections, see nature.com/documents/nr-reporting-summary-flat.pdf

Life sciences study design

All studies must disclose on these points even when the disclosure is negative.

Sample size	We analyzed the whole institutional database of female breast cancer and we selected a very rare population of patients with synchronous bilateral breast cancers (sBBCs) treated by neoadjuvant chemotehrapy and with full pre and post NAC frozen biobanked samples available.
Data exclusions	No data were excluded from analyses
Replication	No replication was available as we analyzed data from patients treated in routine care.
Randomization	Randomization was not appplicable as we analyzed data from patients diagnosed with sBBCs and treated in routine care.
Blinding	Pathologist was blind to response to treatment and prognostic data when retrospectively evaluating immune infiltration (stromal and intra tumoral TILs).

Reporting for specific materials, systems and methods

We require information from authors about some types of materials, experimental systems and methods used in many studies. Here, indicate whether each material, system or method listed is relevant to your study. If you are not sure if a list item applies to your research, read the appropriate section before selecting a response.

Materials & experimental systems

n/a	Involved in the study	
☒	☐	Antibodies
☒	☐	Eukaryotic cell lines
☒	☐	Palaeontology and archaeology
☒	☐	Animals and other organisms
☐	☒	Clinical data
☒	☐	Dual use research of concern

Methods

n/a	Involved in the study	
☒	☐	ChIP-seq
☒	☐	Flow cytometry
☒	☐	MRI-based neuroimaging

Clinical data

Policy information about clinical studies

All manuscripts should comply with the ICMJE guidelines for publication of clinical research and a completed CONSORT checklist must be included with all submissions.

Clinical trial registration	N/A
Study protocol	N/A
Data collection	We identified a cohort of 17575 female patients with non-metastatic breast cancer treated at the Institut Curie (Paris and Saint Cloud, France) between 2005 and 2015 in the institutional database (CNIL number 1766392 – v1, data collection and storage in REDCap* 12.4.14 and MACRO version 3*). Patients were treated according to local guidelines. When indicated, chemotherapy was administered in a neoadjuvant or adjuvant setting, endocrine therapy was indicated in the case of positivity for hormone receptor and according to prognostic factors, and patients with HER2-positive tumors received neoadjuvant and/or adjuvant trastuzumab from 2007 onwards. This study was approved by the Breast Cancer Study Group and by the Institutional Review Board of Institut Curie and was conducted in accordance with institutional and ethical rules regarding research on tissue specimens and patients.
Outcomes	N/A
